# Plant-derived compounds strigolactone GR24 and pinosylvin activate SIRT1 and enhance glucose uptake in rat skeletal muscle cells

**DOI:** 10.1038/s41598-017-17840-x

**Published:** 2017-12-14

**Authors:** Shalem Modi, Nagendra Yaluri, Tarja Kokkola, Markku Laakso

**Affiliations:** 1 Institute of Clinical Medicine, Internal Medicine, University of Eastern Finland, 70210 Kuopio, Finland; 2 Department of Medicine, Kuopio University Hospital, 70210 Kuopio, Finland

## Abstract

Insulin resistance is a characteristic finding in hyperglycaemia and type 2 diabetes. SIRT1 is a NAD^+^ dependent deacetylase that plays a central role in glucose homeostasis and energy metabolism. SIRT1 activators, including plant polyphenols such as resveratrol, improve insulin sensitivity in skeletal muscle tissue. We hypothesised that the novel plant-derived compounds, strigolactone and pinosylvin, beneficially enhance SIRT1 function, insulin signalling, glucose uptake, and mitochondrial biogenesis in skeletal muscle cells. Rat L6 skeletal muscle myotubes were treated with strigolactone analogue GR24 and pinosylvin. Resveratrol was included in experiments as a reference compound. We measured the effects of these compounds on SIRT1 function, insulin signalling, glucose uptake, mitochondrial biogenesis and gene expression profiles. Strigolactone GR24 upregulated and activated SIRT1 without activating AMPK, enhanced insulin signalling, glucose uptake, GLUT4 translocation and mitochondrial biogenesis. Pinosylvin activated SIRT1 *in vitro* and stimulated glucose uptake through the activation of AMPK. The regulation of SIRT1 by strigolactone GR24 and the activation of AMPK by pinosylvin may offer novel therapeutic approaches in the treatment of insulin resistance in skeletal muscle.

## Introduction

Type 2 diabetes, characterised by impaired insulin secretion and insulin resistance, is an important global health problem^1^. Skeletal muscle is the predominant tissue for insulin-stimulated glucose uptake. SIRT1 (Silent information regulator type 1) is a NAD^+^ dependent deacetylase and a key metabolic sensor of energy level changes, and it plays a central role in calorie restriction^2^. SIRT1 activation mimics calorie restriction^3^, and has beneficial effects in age-related diseases, such as cardiovascular, neurodegenerative and metabolic diseases, including type 2 diabetes^4^.

SIRT1 activation controls glucose and lipid metabolism in several tissues^5–8^, improves insulin sensitivity^9,10^, stimulates mitochondrial biogenesis^11^, and decreases obesity-induced inflammation in macrophages^9^. SIRT1 regulates energy metabolism by interacting with AMP-activated protein kinase (AMPK)^6,11,12^, a master cellular energy sensor controlling metabolic homeostasis and cell survival during stress, and peroxisome proliferator-activated receptor gamma coactivator 1-alpha (PGC1α), a major regulator of mitochondrial biogenesis^11^. Our previous studies have shown that SIRT1 expression is correlated with insulin sensitivity and mitochondrial function^13^, and that activation of SIRT1 prevents the development of metabolic syndrome *via* the deacetylation and activation of PGC1α^14^. SIRT1 activators, including plant polyphenols such as resveratrol, have beneficial effects on glucose homeostasis and insulin sensitivity in animal models^15^.

Strigolactones are plant hormones that enhance beneficial symbiosis of the plants with mycorrhizal fungi where they promote mitochondrial biogenesis, hyphal branching and ATP production^16,17^. Strigolactones have beneficial effects in cancer^18,19^, but their effects on glucose metabolism have not been investigated in mammalian cells. Pinosylvin, a stilbenoid polyphenol whose structure closely resembles resveratrol, is a natural compound found in high concentrations in the *Pinus* species. Pinosylvin has anti-bacterial, anti-fungal and anti-cancer properties^20,21^, but its effects in metabolically relevant tissues have not been previously investigated in detail^22^. We hypothesised that strigolactone and pinosylvin have beneficial effects on glucose and energy metabolism, and therefore we investigated the effects of strigolactone analogue GR24 and pinosylvin on SIRT1 function, insulin sensitivity, mitochondrial biogenesis and gene expression in differentiated skeletal muscle cells.

## Results

### GR24 and pinosylvin enhance glucose uptake and stimulate GLUT4 translocation

**Figure 1:**
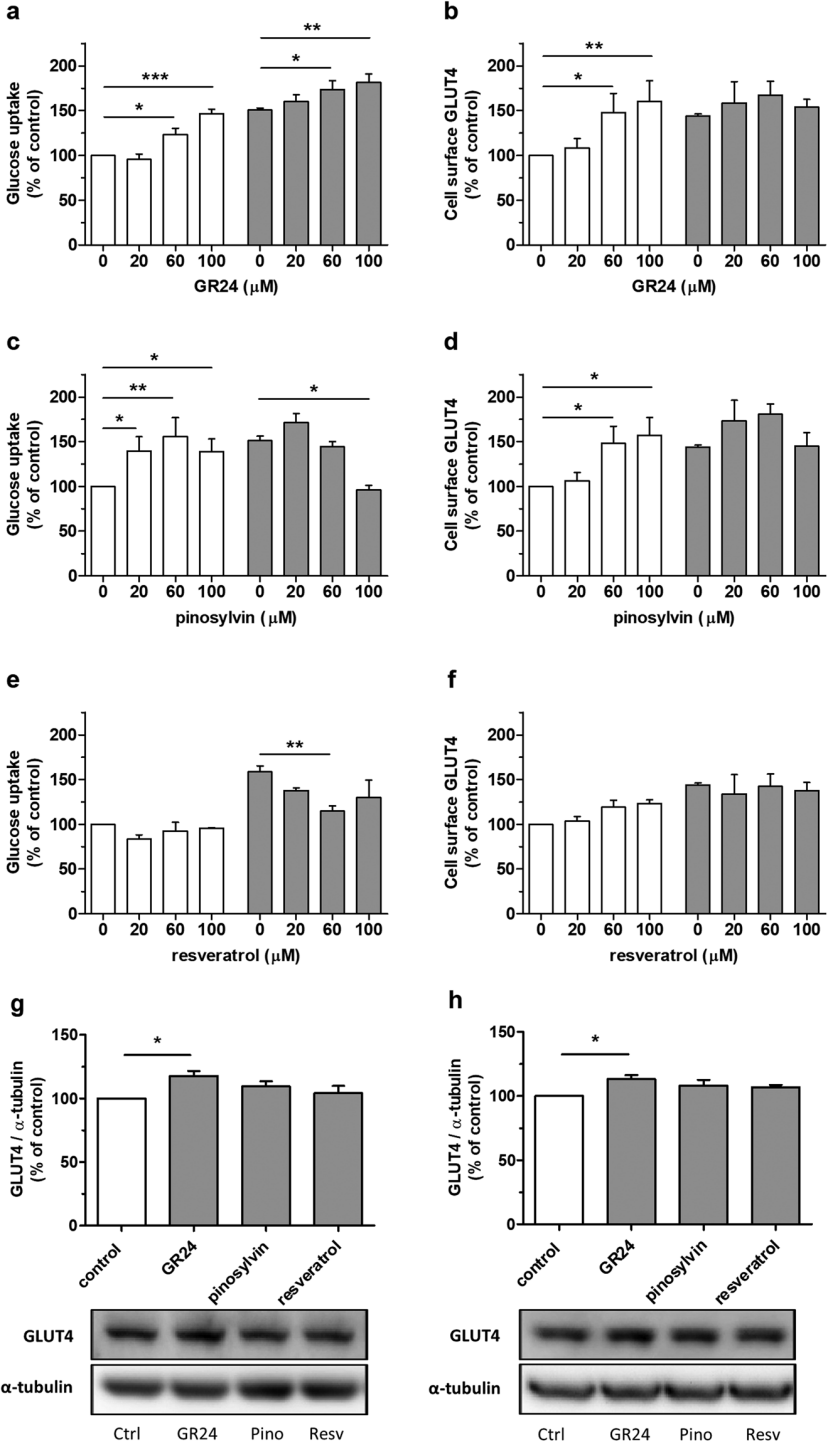
GR24 and pinosylvin stimulate glucose uptake and GLUT4 translocation, and GR24 upregulates GLUT4 expression. (**a**–**f**) L6 myotubes were treated with 0–100 µM test compounds for 6 h without insulin (white bars) or with 100 nM insulin (grey bars). (**a**,**c**,**e**) Glucose uptake was measured using [^3^H]2-deoxyglucose uptake assay and (**b**,**d**,**f**) cell surface GLUT4 was measured using in-cell western assay. (**g**,**h**) L6 myotubes were treated with 60 µM test compounds (grey bars) or with DMSO (white bar) for 24 h in media containing 5.5 mM glucose (**g**) or 16.7 mM glucose (**h**). (**g**,**h**) After western blotting, GLUT4 expression was quantified and normalised with α-tubulin detected from the same membranes after stripping and reprobing. Representative western blots are shown below. Full-length blots are presented in Supplementary information. (**a**–**h**) Bars represent the mean ± SEM of three to four independent experiments. *p < 0.05, **p < 0.01 and ***p < 0.001 between the indicated groups.

GR24 enhanced basal and insulin-stimulated glucose uptake dose-dependently, and significantly at 60 µM and 100 µM concentrations (Fig. 1a). GR24 also stimulated GLUT4 translocation significantly at 60 µM and 100 µM concentrations in basal conditions, but its effect did not reach statistical significance in insulin-stimulated conditions (Fig. 1b). Pinosylvin enhanced basal glucose uptake significantly at all tested concentrations, with maximal effect at 60 µM, but at 100 µM it inhibited insulin-stimulated glucose uptake (Fig. 1c). Pinosylvin stimulated GLUT4 translocation at 60 µM and 100 µM concentrations only in basal conditions (Fig. 1d). Resveratrol did not affect basal glucose uptake, but had a significant inhibitory effect on insulin-stimulated glucose uptake at 60 µM (Fig. 1e). Resveratrol had no significant effect on GLUT4 translocation (Fig. 1f). The results of GLUT4 in-cell western assays were confirmed by visualizing cell surface GLUT4 levels with fluorescent microscopy. The microscopic images showed that GR24 and pinosylvin stimulated GLUT4 translocation (Supplementary Fig. 2).

### GR24 upregulates GLUT4 protein

L6 myotubes were treated with 60 µM GR24, pinosylvin and resveratrol for 24 hours, and the lysates were analysed by western blotting. The treatments were tested to elicit no or minimal cytotoxicity in the myotubes (Supplementary Fig. 3). GR24 significantly increased GLUT4 expression at both normal (5.5 mM) and high glucose levels (16.7 mM) (Fig. 1g,h).

### GR24 upregulates and activates SIRT1 in skeletal muscle cells, whereas pinosylvin stimulates AMPK phosphorylation and activates SIRT1 *in vitro*

**Figure 2:**
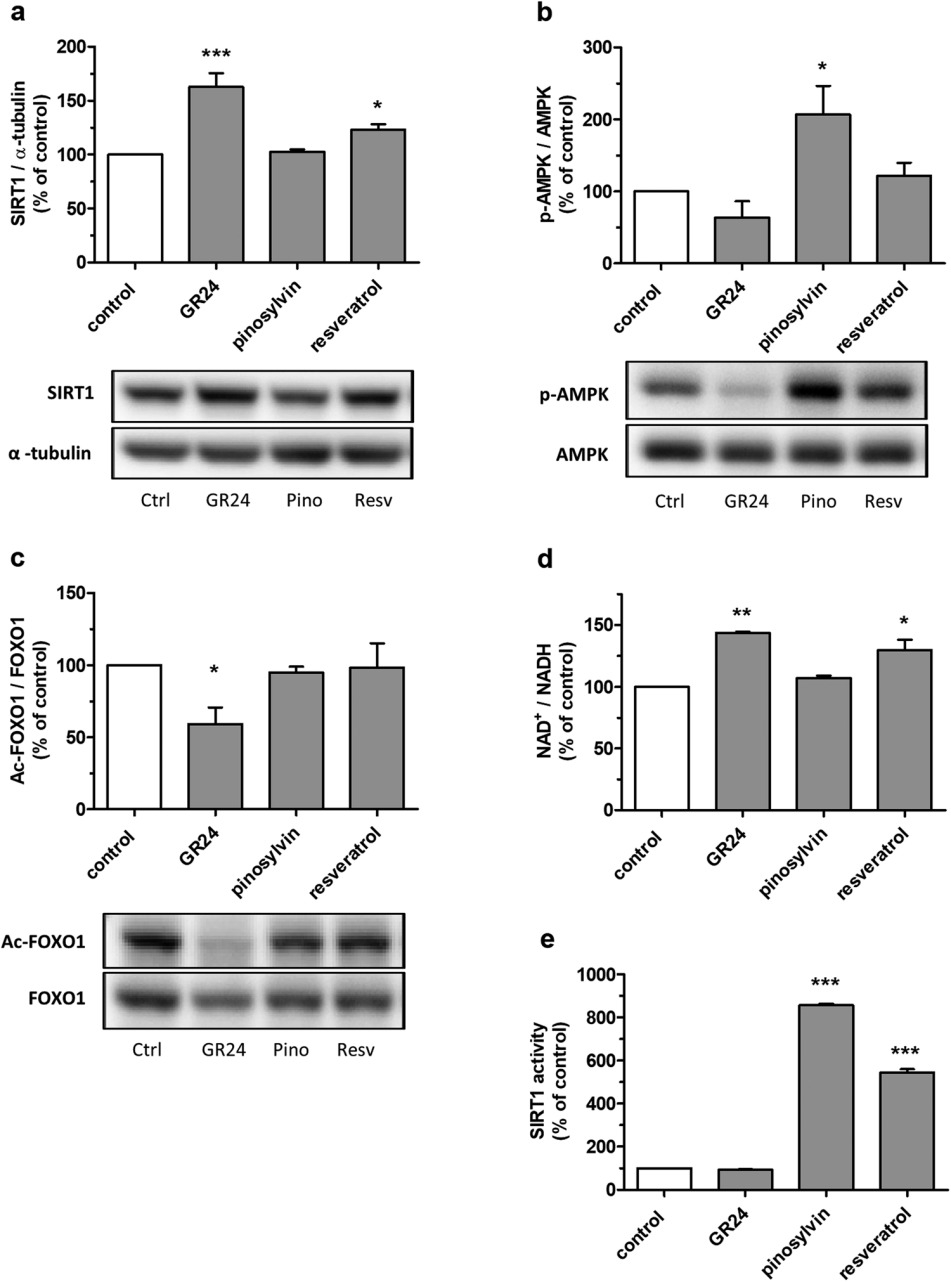
GR24 upregulates and activates SIRT1 in skelatal muscle cells, whereas pinosylvin stimulates AMPK phosphorylation and activates SIRT1 *in vitro*. (**a**–**c**) L6 myotubes were treated with 60 µM test compounds (grey bars) or with DMSO (white bar) for 24 h in media containing 5.5 mM glucose. (**a**) SIRT1 expression was analysed by western blotting; bars represent the quantification results of SIRT1 normalised with α-tubulin. (**b**) AMPK phosphorylation was analysed by western blotting; bars represent the quantification results of phospho-AMPK normalised with total AMPK. (**c**) Western blotting was used to analyze the acetylation level of FOXO1; bars represent the quantification results of acetylated FOXO1 normalised with total FOXO1. (**a**–**c**) Bars represent the mean ± SEM of three independent experiments. *p < 0.05 and ***p < 0.001 vs control. Representative western blots are shown below. Full-length blots are presented in Supplementary information. Proteins in lower panels were detected from the same membranes after stripping and reprobing. (**d**) NAD^+^/NADH ratios were quantified spectrophotometrically from L6 myotube cell lysates after treatment with 60 µM test compounds (grey bars) or with DMSO (white bar) for 24 h. Bars represent the mean ± SEM of two independent experiments. *p < 0.05 and **p < 0.01 vs control. (**e**) Recombinant SIRT1 activity in Fluor de Lys *in vitro* activity assay. The compounds were used at 100 µM concentration. Bars represent the mean ± SEM of six independent experiments. ***p < 0.001 vs control.

GR24 and resveratrol treatments enhanced significantly the protein expression of SIRT1, whereas pinosylvin had no significant effect on SIRT1 (Fig. 2a). Pinosylvin stimulated AMPK phosphorylation, but GR24 and resveratrol had no significant effect on AMPK (Fig. 2b). FOXO1 acetylation decreased in GR24-treated cells, whereas pinosylvin and resveratrol treatment did not significantly change the acetylation status of FOXO1 (Fig. 2c). At high glucose conditions (16.7 mM), these findings on SIRT1, AMPK and acetylated FOXO1 were essentially similar (Supplementary Fig. 4a–c). GR24 and resveratrol stimulated the production of NAD^+^, which is an essential co-substrate of SIRT1, and increased the NAD^+^/NADH ratio in myotubes treated with 60 µM compounds for 24 h (Fig. 2d). Pinosylvin did not have an effect on the NAD^+^/NADH ratio (Fig. 2d). The test compounds had no significant effect on SIRT1 activity in cell lysates tested with Fluor de Lys assay (Supplementary Fig. 5a).

The ability of the compounds to activate recombinant SIRT1 enzyme was tested in two different cell-free *in vitro* activity assays. In Fluor de Lys assay which uses the p53-based fluorescent peptide substrate, 100 µM pinosylvin and resveratrol strongly activated SIRT1, but GR24 showed no effect (Fig. 2e). In further Fluor de Lys *in vitro* assays, pinosylvin was found to stimulate SIRT1 in a dose-dependent manner, with EC_50_ value of 116.8 ± 7.5 µM and E_max_ value of 1976 ± 75% (Supplementary Fig. 5b). In another *in vitro* activity assay, SIRTainty, GR24 did not activate SIRT1 with either of the two tested substrates. Pinosylvin and resveratrol activated SIRT1 towards the p53-based Fluor de Lys fluorescent peptide substrate, but had no effect on the deacetylation of a non-labelled peptide substrate supplied with the commercial SIRTainty assay kit (proprietary sequence) (Supplementary Fig. 5c).

### GR24 stimulates mitochondrial biogenesis and ATP production

**Figure 3:**
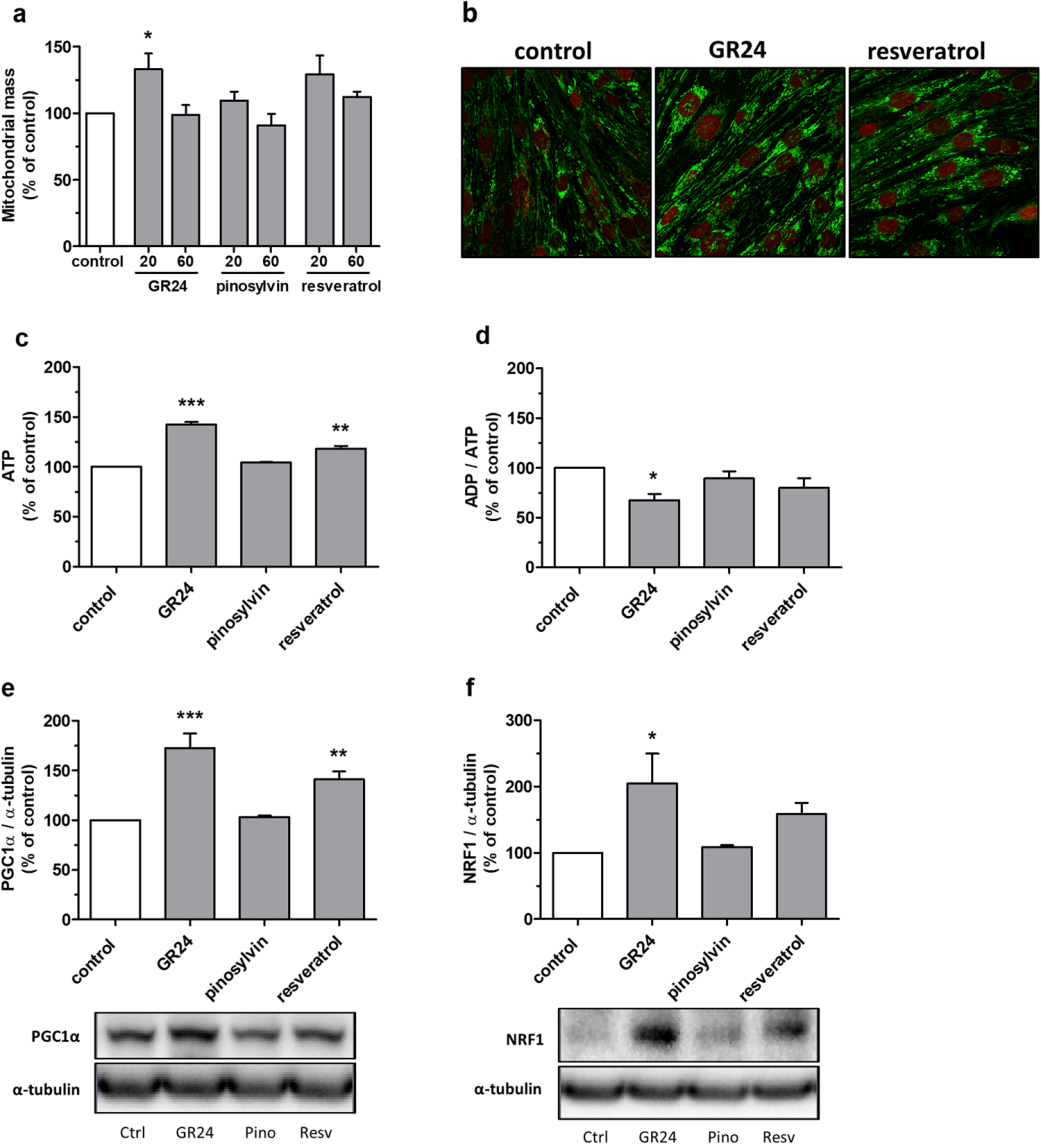
GR24 stimulates mitochondrial biogenesis, upregulates mitochondrial regulators and increases ATP production. (**a**) Plate reader assay of mitochondrial mass. L6 myotubes were treated with 20 µM or 60 µM test compounds (grey bars) or with DMSO (white bar) for 24 h. The mitochondria were stained with MitoTracker Green FM and the fluorescence was quantified with a plate reader. Bars represent the mean ± SEM of three independent experiments. *p < 0.05 vs control. (**b**) Fluorescent microscopic images of L6 myotubes treated with 20 µM test compounds or with DMSO for 24 h. The mitochondria were visualized with MitoTracker Green FM (green) and the nuclei with DRAQ5 (red). (**c**–**f**) L6 myotubes were treated with 60 µM test compounds (grey bars) or with DMSO (white bar) for 24 h. (**c**) ATP concentration and (**d**) ADP/ATP ratio were quantified spectrophotometrically. (**c**,**d**) Bars represent the mean ± SEM of two independent experiments. *p < 0.05, **p < 0.01 and ***p < 0.001 vs control. (**e**) PGC1α expression was analysed by western blotting; bars represent the quantification results of PGC1α normalised with α-tubulin. (**f**) NRF1 expression was analysed by western blotting; bars represent the quantification results of NRF1 normalised with α-tubulin. (**e**,**f**) Bars represent the mean ± SEM of three independent experiments. *p < 0.05, **p < 0.01 and ***p < 0.001 vs control. Representative western blots are shown below. Full-length blots are presented in Supplementary information. α-tubulin was detected from the same membranes after stripping and reprobing.

Mitochondrial mass increased significantly in 20 µM GR24-treated L6 myotubes (Fig. 3a). This finding was confirmed by confocal imaging of mitochondria, showing enhanced mitochondrial staining (Fig. 3b). Pinosylvin did not show any effect on mitochondrial biogenesis, but 20 µM resveratrol exhibited a trend to induce mitochondrial biogenesis. Treatment of L6 myotubes with 60 µM GR24 resulted in elevated production of ATP (Fig. 3c) and a decrease in the ADP/ATP ratio (Fig. 3d). Resveratrol stimulated ATP production, but pinosylvin treatment had no effect on ATP level or the ADP/ATP ratio. In western blotting analyses from L6 myotube lysates treated with 60 µM test compounds for 24 h at normal glucose (5.5 mM), PGC1α expression was found to be increased by GR24 and resveratrol treatments (Fig. 3e). Respiratory factor 1 (NRF1) expression increased by GR24 treatment (Fig. 3f). Pinosylvin treatment did not affect PGC1α or NRF1 expression. Similar results for PGC1α and NRF1 expression were obtained in high glucose conditions (16.7 mM, Supplementary Fig. 4d,e).

### Microarray analysis of transcripts relevant in SIRT1 expression, insulin signalling, mitochondrial function and metabolism

Microarrays were applied to compare transcriptomes in L6 myotubes exposed to 60 µM GR24, pinosylvin and resveratrol for 24 hours to transcriptomes in control-treated myotubes. There were a total of 16,153 differentially regulated transcripts (9,838 up- and 6,315 downregulated) to the GR24 treatment (Supplementary Fig. 6). The raw data have been deposited in the Gene Expression Omnibus (GEO) database with accession no. GSE90833.

Among the individual genes, GR24 activated the transcription of SIRT1 as well as sirtuins SIRT3 and SIRT6, NAD^+^ synthesizing enzyme nicotinamide phosphoribosyltransferase (NAMPT), and transcription factors (Arntl, Creb1, and Myc) which have been shown to positively regulate SIRT1 (Table 1). Moreover, several genes involved in insulin signalling were upregulated, including insulin receptor, insulin receptor substrate 1 (IRS-1) and phosphatidylinositol-3-kinase (PI3K) (Table 2). AKT2 was downregulated, but its activating kinase 3-phosphoinositide dependent protein kinase-1 (PDPK1) was upregulated (Table 2). GLUT4 transcript was downregulated, whereas the expression of GLUT4 protein degradation inhibitor calpastatin was upregulated (Table 2). GR24 also activated the transcripts of several genes encoding mitochondrial proteins (Table 2). The effects of pinosylvin and resveratrol on the transcripts were less clear (Tables 1 and 2). Both pinosylvin and resveratrol upregulated SIRT3 but did not have significant effects on NAMPT transcription.

**Table 1:** Selected up- and downregulated transcripts of sirtuins, Nampt and transcription factors in L6 myotubes after 24 h treatment with 60 μM test compounds.

**Gene Symbol**	**Accession**	**GR24**		**Pinosylvin**		**Resveratrol**	
		**Fold change**	**Adj**. ***p*** **value**	**Fold change**	**Adj**. ***p*** **value**	**Fold change**	**Adj**. ***p*** **value**
Sirt1	ENSRNOT 00000000427	1.34	**1.08 × 10** ^**−6**^	*0.83*	1.43 × 10^**−**1^	1.06	6.58 × 10^**−**1^
Sirt3	ENSRNOG 00000013828	1.40	**6.53 × 10** ^**−5**^	1.25	**5.29 × 10** ^**−3**^	1.20	**1.39 × 10** ^**−2**^
Sirt6	NM_001031649	1.28	**1.45 × 10** ^**−3**^	1.13	1.16 × 10^**−**1^	1.09	2.41 × 10^**−**1^
Nampt	NM_177928	1.67	**3.75 × 10** ^**−4**^	0.93	5.66 × 10^**−**1^	1.06	6.88 × 10^**−**1^
Arntl	NM_024362	1.52	**1.55 × 10** ^**−4**^	1.17	9.03 × 10^**−**2^	1.26	**2.27 × 10** ^**−2**^
Creb1	NM_134443	1.66	**1.32 × 10** ^**−5**^	*0.88*	1.80 × 10^**−**1^	*0.88*	1.20 × 10^**−**1^
Myc	NM_012603	2.28	**6.06 × 10** ^**−8**^	1.41	**2.49 × 10** ^**−4**^	1.56	**5.36 × 10** ^**−5**^

Sirt1, sirtuin1; Sirt3, sirtuin 3; Sirt6, sirtuin 6; Nampt, nicotinamide phosphoribosyltransferase; Arntl, aryl hydrocarbon receptor nuclear translocator-like; Creb1, cAMP responsive element binding protein; Myc, myelocytomatosis oncogene; Fold change shown for treated vs control groups, positive values reflect upregulation and negative values (shown in italics) reflect downregulation; Adj. *p* value, *p* value after Benjamini-Hochberg FDR correction, significance threshold set at *p* < 0.05, significant values shown in bold.

**Table 2:** Selected up- and downregulated transcripts involved in insulin signalling and mitochondrial function in L6 myotubes after 24 h treatment with 60 μM test compounds.

**Gene Symbol**	**Accession**	**GR24**		**Pinosylvin**		**Resveratrol**	
		**Fold change**	**Adj**. ***p*** **value**	**Fold change**	**Adj**. ***p*** **value**	**Fold change**	**Adj**. ***p*** **value**
Insr	NM_017071	1.98	**1.08 × 10** ^**−6**^	1.03	7.75 × 10^**−**1^	1.14	9.99 × 10^**−**2^
Irs1	NM_012969	1.35	**1.30 × 10** ^**−2**^	1.11	4.23 × 10^**−**1^	1.33	**1.30 × 10** ^**−2**^
Pik3c3	NM_022958	1.68	**9.20 × 10** ^**−7**^	1.02	7.55 × 10^**−**1^	1.00	9.72 × 10^**−**1^
Pdpk1	NM_031081	1.66	**9.30 × 10** ^**−5**^	*0.99*	9.41 × 10^**−**1^	1.07	5.61 × 10^**−**1^
Akt2	NM_017093	*0.85*	**3.28 × 10** ^**−3**^	*0.99*	8.63 × 10^**−**1^	*0.86*	**1.36 × 10** ^**−2**^
Slc2a4	NM_012751	*0.45*	**2.08 × 10** ^**−7**^	1.22	**8.71 × 10** ^**−3**^	*0.96*	6.30 × 10^**−**1^
Cast	NM_053295	1.76	**3.26 × 10** ^**−4**^	*0.90*	5.91 × 10^**−**2^	1.14	3.60 × 10^**−**1^
Cs	NM_130755	1.47	**5.35 × 10** ^**−6**^	1.06	3.21 × 10^**−**1^	1.13	**3.50 × 10** ^**−2**^
Immt	NM_001034928	1.28	**7.59 × 10** ^**−4**^	1.04	5.76 × 10^**−**1^	1.00	9.71 × 10^**−**1^
Mrpl2	NM_001034136	1.79	**6.14 × 10** ^**−7**^	1.11	9.45 × 10^**−**2^	1.19	**1.14 × 10** ^**−2**^
Pc	NM_012744	1.64	**4.98 × 10** ^**−7**^	1.34	**1.72 × 10** ^**−4**^	1.59	**1.03 × 10** ^**−5**^
Sdha	NM_130428	1.12	**1.23 × 10** ^**−2**^	1.08	1.31 × 10^**−**1^	1.00	9.54 × 10^**−**1^
Tfam	NM_031326	1.79	**1.58 × 10** ^**−6**^	1.11	1.46 × 10^**−**1^	1.14	6.10 × 10^**−**2^

Insr, insulin receptor; Irs1, insulin receptor substrate 1; Pik3c3, phosphatidylinositol 3-kinase catalytic subunit type 3; Pdpk1, 3-phosphoinositide dependent protein kinase 1; Akt2, AKT serine/threonine kinase 2; Slc2a4, solute carrier family 2 member 4 (GLUT4); Cast, calpastatin; Cs, citrate synthase; Immt, inner membrane mitochondrial protein; Mrpl2, mitochondrial ribosomal protein L2; Pc, pyruvate carboxylase; Sdha, succinate dehydrogenase subunit A; Tfam, transcription factor A mitochondrial; Fold change shown for treated vs control groups, positive values reflect upregulation and negative values (shown in italics) reflect downregulation; Adj. *p* value, *p* value after Benjamini-Hochberg FDR correction, significance threshold set at *p* < 0.05, significant values shown in bold.

Heat map and clustering analysis of the transcripts of the “Mitochondrion organization and biogenesis” and “Insulin receptor signalling pathway” Gene Ontology (GO) gene sets are presented in Supplementary Figures 7 and 8. The clustering analysis shows clearly that GR24-treated samples had different expression patterns and formed a separate cluster in all of the gene sets, showing increased expression for most of the individual genes. By contrast in “mitochondrion organization and biogenesis” GO, pinosylvin and resveratrol clustered together with the control group, indicating that these compounds did not have significant effects on the transcripts of the gene set investigated.

## Discussion

**Figure 4:**
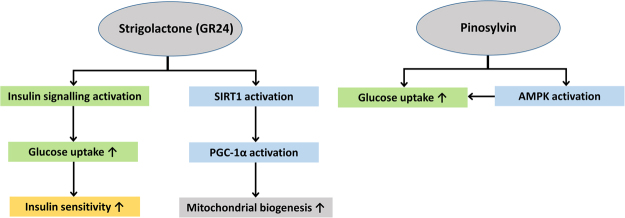
Summary of the most important effects of GR24 and pinosylvin on glucose and energy metabolism in skeletal muscle cells. Observed increase is indicated by an upwards arrow (↑).

We hypothesised that the two plant-derived compounds, GR24 and pinosylvin, may have beneficial effects on glucose and energy metabolism and therefore we investigated their effects on SIRT1 function, glucose uptake (insulin sensitivity), mitochondrial biogenesis, and gene expression of key regulators of these pathways in L6 skeletal muscle myotubes. Resveratrol was included in the experiments as a reference compound. Our study reports several novel findings: 1. GR24 and pinosylvin stimulated skeletal muscle glucose uptake and GLUT4 translocation, 2. GR24 upregulated and activated SIRT1 in skeletal muscle cells without activating AMPK, whereas pinosylvin activated AMPK, 3. GR24 stimulated mitochondrial biogenesis and transcription of mitochondrial proteins, enhanced ATP and NAD^+^ production and upregulated PGC1α and NRF1 (Findings summarised in Fig. 4).

GR24 and pinosylvin significantly stimulated glucose uptake in L6 myotubes and insulin-stimulated cells in basal conditions, but resveratrol failed to increase glucose uptake. Previous studies have reported conflicting findings, one study showed that resveratrol stimulated skeletal muscle glucose uptake *via* the activation of both SIRT1 and AMPK^23^, whereas other studies reported no effect^24^, or an inhibitory effect^25^. Differences in experimental conditions of these studies may explain conflicting findings. GLUT4 is the principal glucose transporter in the skeletal muscle and insulin stimulates glucose uptake by promoting GLUT4 translocation to the cell surface^26^. We did not observe a significant effect on GLUT4 translocation by resveratrol, whereas GR24 and pinosylvin significantly promoted GLUT4 translocation, in agreement with the effect of these compounds on glucose uptake.

Possible mechanisms of GR24-stimulated glucose uptake could be the upregulation of GLUT4 at the protein level and the activation of insulin signalling pathway. GR24 treatment downregulated GLUT4 transcript expression, suggesting that GR24 seems to conserve GLUT4 from degradation. The mechanism could be the upregulation of calpastatin, an inhibitor of GLUT4 degrading enzyme calpain 2^27^. Calpastatin overexpression results in increased GLUT4 protein and decreased GLUT4 mRNA in the skeletal muscle^27^. GR24 also positively stimulated the insulin signalling pathway by upregulating insulin receptor, IRS-1 and PI3K. The transcription of AKT2 was decreased, but its activating kinase PDPK1 was upregulated, which increases the activity of AKT2.

SIRT1 and AMPK form a positive feedback cycle in many experimental conditions, and resveratrol produces its effects on SIRT1 by activating first AMPK^12,28^. In order to investigate the roles of SIRT1 and AMPK on the stimulation of glucose uptake by GR24 and pinosylvin, we analysed the levels of SIRT1 and phosphorylated (active) AMPK, and measured cellular SIRT1 activity and NAD^+^ levels. GR24 significantly upregulated SIRT1 protein expression without having a significant effect on AMPK activation. Conversely, pinosylvin activated AMPK but did not modulate the expression of SIRT1, confirming the previously reported AMPK-activating effect of pinosylvin^29^. Resveratrol did not activate AMPK in our study, and it only slightly upregulated SIRT1 in normal glucose conditions. Most importantly, GR24 increased SIRT1 deacetylase activity towards its endogenous substrate FOXO1, and decreased acetylated FOXO1 levels in cell lysates, but did not activate SIRT1 when cell lysates were tested in Fluor de Lys *in vitro* assay. Different SIRT1 activators and activating pathways exert selective effects on deacetylation of various substrates, which may explain our results^30,31^. Pinosylvin and resveratrol did not significantly change the acetylation of FOXO1 or modulate deacetylation of the p53-based substrate in cell lysates, indicating that they did not activate cellular SIRT1 in our experimental setting.

We showed that resveratrol and pinosylvin strongly activated deacetylation of the fluorescently labelled p53-based substrate by recombinant SIRT1 in Fluor de Lys *in vitro* assays, but GR24 did not have this effect. Pinosylvin activated SIRT1 at micromolar concentrations with almost 20-fold maximum stimulation. As the labelled substrates can cause artefact results^32^, we also tested the compounds in SIRTainty assay, which allows for the use of various unlabelled substrates^30^. GR24 failed to modulate the activity of SIRT1 also in this assay. Both pinosylvin and resveratrol activated SIRT1 only towards the p53-based fluorescent peptide substrate, but not towards a non-labelled peptide substrate in the SIRTainty assay. This confirms that the activation of SIRT1 by resveratrol is substrate sequence-selective^30,31^ and reveals that pinosylvin resembles resveratrol in this respect. Taken together, these results indicate that GR24 is not activating SIRT1 in *in vitro* assays, but is able to boost cellular NAD^+^ levels and stimulate deacetylase activity of SIRT1 towards its endogenous substrate protein. In contrast, pinosylvin directly activates SIRT1 in some *in vitro* assay conditions, but does not modulate NAD^+^ or activate SIRT1 in skeletal muscle cells.

We found in our transcriptome analysis that GR24 upregulated SIRT1 expression in agreement with the upregulation of SIRT1 at the protein level. The increase in NAMPT transcription may explain the increase in NAD^+^ production. Our microarray results showed an upregulation of several transcription factors (Arntl, Creb1, Myc) which positively regulate SIRT1, providing possible mechanisms for upregulation of SIRT1. Pinosylvin did not regulate SIRT1 or NAMPT transcript levels and had less effect on SIRT1 regulating transcription factors. This is in line with the fact that pinosylvin treatment did not affect the levels of SIRT1 protein or NAD^+^.

Mitochondrial function is essential for the oxidation of lipids and carbohydrates in skeletal muscle. Reduced rate of mitochondrial oxidative phosphorylation has been reported in skeletal muscle of patients with type 2 diabetes^33^, and proteomic analysis of insulin-resistant muscle has confirmed a lower abundance of mitochondrial proteins^34^. Skeletal muscle insulin resistance due to mitochondrial dysfunction can be attenuated by overexpression of SIRT1^35^, and our previous study showed that resveratrol improves mitochondrial function in skeletal muscle through activating transcription factor coactivator PGC1α^14^. We found that resveratrol upregulated PGC1α and increased the production of ATP in L6 myotubes, but the stimulation of mitochondrial biogenesis by resveratrol did not reach statistical significance. GR24 stimulated mitochondrial biogenesis and upregulated PGC1α and NRF1, the two of the most important mitochondrial biogenesis factors, whereas pinosylvin did not have this effect. Treatment with GR24 enhanced the production of ATP and increased the NAD^+^/NADH ratio, which is in agreement with enhanced oxidative phosphorylation in skeletal muscle mitochondria^36^. Strigolactones have previously been shown to stimulate mitochondrial biogenesis and ATP production in mycorrhizal fungi^16,17^. Our study is the first to show that the same mechanism is working in skeletal muscle cells.

GR24 upregulated transcripts of several mitochondrial proteins, including mitochondrial transcription factor A (TFAM), an important activator of mitochondrial transcription, and citrate synthase, a marker of mitochondrial density in the skeletal muscle^37^. In general, based on the heat maps and clustering analyses of the gene sets, a clear upregulation of genes involved in mitochondrial function was evident after GR24 treatment. This further confirms the stimulation of mitochondrial biogenesis by GR24 treatment. On the other hand, pinosylvin did not show any distinct effect on the transcripts regulating mitochondrial function, which is in accordance with our other findings. Resveratrol upregulated citrate synthase and some other genes regulating mitochondrial function, as previously reported^11^.

In summary, our study extends the knowledge on pinosylvin showing its potential in promoting glucose uptake in skeletal muscle cells and activating SIRT1 towards different substrates *in vitro*. Most importantly, our findings introduce plant hormone strigolactones as a new class of phytochemicals with multiple beneficial effects on insulin sensitivity, mitochondrial biogenesis and cellular SIRT1 activity. Therefore, these compounds may have potential in treatment of insulin resistance and type 2 diabetes.

## Materials and Methods

### Cell culture and differentiation

Rat L6 myoblasts (ATCC, Manassas, VA, USA) were maintained in Dulbecco’s modified Eagle’s medium (DMEM 4.5 g/l glucose, Lonza, Basel, Switzerland) supplemented with 10% FBS (Hyclone, Pasching, Austria), 2 mM L-glutamine (Lonza) and 1% penicillin/streptomycin (Lonza) at +37 °C in a humidified atmosphere of 5% CO_2_. The cultures were regularly checked for the presence of mycoplasma. The cells were seeded in multiwell plates at the density of 2 × 10^4^ cells/cm^2^ one day before the start of the differentiation. The differentiation of myoblasts into myotubes was induced by switching the media into α-MEM (Gibco, Paisley, UK) supplemented with 2% horse serum (Gibco) and 1% penicillin/streptomycin. The differentiation medium was replenished every 48 h and the cells were treated with test compounds at 6–7 days of differentiation for 24 h in differentiation media, unless otherwise stated. The test compounds, strigolactone analogue GR24 ((3E,3aR,8bS)-3-[[(2 S)-4-methyl-5-oxo-2H-furan-2-yl] oxymethylidene]-4,8b-dihydro-3aH-indeno[1,2-b]furan-2-one, Chiralix, Nijmegen, Netherlands), pinosylvin (3,5-dihydroxy-*trans*-stilbene, Sequoia, Pangbourne, UK) and resveratrol (3,4′,5-trihydroxy-*trans*-stilbene, Sigma, Schnelldorf, Germany), were dissolved and diluted in DMSO. The control samples were treated with equivalent concentration of DMSO. The chemical structures of the test compounds are shown in Supplementary Fig. 1.

### Deoxyglucose uptake

Deoxyglucose uptake measurements were performed as previously described^38^. Briefly, the L6 myotubes were differentiated in 24-well plates and exposed to the test compounds or equal concentration of DMSO for 6 h in duplicate wells, in differentiation media ± 100 nM human insulin (Sigma). The cells were washed with phosphate buffered saline (PBS) and starved for 60 min in glucose-free HEPES-buffered saline (20 mM HEPES, 140 mM NaCl, 5 mM KCl, 2.5 mM MgSO_4_, 1 mM CaCl_2_, pH 7.4) while maintaining the same concentrations of test compounds and insulin. Finally, HEPES-buffered saline containing 10 μM 2-deoxy-D-glucose (Sigma) and 0.5 μCi/μl [^3^H]2-Deoxy-D-glucose (PerkinElmer, Turku, Finland) was added on the myotubes and the uptake reaction was allowed to proceed for 15 min at room temperature. The uptake reaction was stopped by washing the cells thrice with ice-cold PBS. The cells were lysed in 0.5 M NaOH and the radioactivity in the lysates was counted by liquid scintillation (Wallac 1414 WinSpectral, Wallac, Turku, Finland). The protein content of the lysates was measured with BCA Protein Assay (Pierce, Rockford, IL, USA).

### In-cell western GLUT4 translocation assay

Cell surface GLUT4 levels were assessed by In-Cell western using the Odyssey Infrared Imaging System (LI-COR Biosciences, Cambridge, UK) with modifications of a previously reported protocol^39^. L6 myotubes were differentiated in 96-well plates and exposed to the test compounds for 6 h in triplicate wells, in differentiation media ± 100 nM human insulin (Sigma). The cells were briefly washed with PBS and fixed with 4% (w/v) paraformaldehyde in PBS for 10 min at room temperature. After washing, nonspecific binding was blocked with blocking solution (DMEM containing 10% FCS) for 30 min at room temperature. Cells were then incubated with 1:100 diluted goat GLUT4 antibody (sc-1606, Santa Cruz, Heidelberg, Germany) in blocking solution overnight at +4 °C. The cells were washed and incubated with Alexa Fluor 680 labelled 1:250 diluted donkey anti-goat antibody (ab175776, Abcam, Cambridge, UK) in blocking solution for 60 min at +37 °C. After extensive washing, the plates were dried and scanned on the Odyssey Infrared Imaging System on the 700 nm channel. The relative intensities of GLUT4 signal were normalized with the protein content of the cells measured with BCA Protein Assay.

Cell surface GLUT4 levels were also visualized with fluorescent microscopy. L6 myotubes were differentiated in 8-well chamber slides (Ibidi, Munich, Germany). The cells were treated and stained as described above for the 96-well plates. As a control, the primary antibody was omitted during the staining procedure to detect the non-specific signal arising from the secondary antibody. The fluorescent images were obtained with Zeiss Axio Observer microscope (40× objective).

### Sulforhodamine B cytotoxicity assay

The L6 cells were differentiated into myotubes in 96-well plates and treated for 6 h or 24 h in differentiation media in triplicate wells. Sulforhodamine B staining and colorimetric assay were performed as previously described^40^. The results were measured with Cytation 3 Multi-Mode Reader (BioTek, Winooski, VT, USA) at 565 nm.

### SIRT1 *in vitro* activity assays

Fluor de Lys SIRT1 activity assays were run in duplicate wells with the materials and methods of the SIRT1 Fluorometric Drug Discovery Kit (BML-AK555, Enzo, Lörrach, Germany), with 25 µM acetylated SIRT1 substrate (BML-KI177, Enzo), 100 µM NAD^+^ and 1 U recombinant SIRT1 (His-tagged human full-length SIRT1, BML-SE239, Enzo). Fluorescence readings were obtained using Cytation 3 Multi-Mode Reader (BioTek) with excitation wavelength 370 nm and emission 460 nm. The activity of the endogenous SIRT1 in cellular lysates was measured according to a previously published Fluor de Lys -based method^41^ using 25 µM acetylated SIRT1 substrate (Enzo), 100 µM NAD^+^ and 20 µg of cellular protein.

SIRTainty Class III HDAC Assay Kit (Millipore, Darmstadt, Germany) was used with two different peptide substrates: a fluorophore-labelled p53-based peptide (BML-KI177, Enzo) and an unlabelled SIRTainty assay peptide with a proprietary sequence (Millipore). The assay employs nicotinamidase to measure nicotinamide generated upon the cleavage of NAD^+^ during the deacetylation of a substrate^42^. Assays were run according to the kit instructions in duplicate wells with 25 µM acetylated peptide substrate, 100 µM NAD^+^, 20 µg/ml nicotinamidase and 1.5 U recombinant SIRT1 (His-tagged human full-length SIRT1, BML-SE239, Enzo). Fluorescence readings were obtained using Victor 1420 Multilabel Counter (PerkinElmer, Turku, Finland) with excitation wavelength 405 nm and emission 460 nm.

### Mitochondrial staining

For confocal microscopy imaging of the mitochondria, the L6 cells were differentiated into myotubes in 8-well chamber slides (Ibidi, Munich, Germany). After treatments, the cells were stained with 100 nM MitoTracker Green FM (Invitrogen, Paisley, UK) for 30 min in serum-free media. Cells were then washed and the nuclei were stained with 2.5 µM DRAQ5 (BioStatus, Shepshed, UK). The fluorescent images were obtained with Zeiss Axio Observer inverted microscope (40× objective) equipped with Zeiss LSM 700 confocal module (Carl Zeiss Microimaging, Jena, Germany). Confocal microscopy settings were set on the intensity of fluorescence from control cells and maintained as such during the acquisition of treated cells. The experiments were repeated at least twice.

The cellular content of mitochondria was quantified by Mitotracker Green FM staining in 24-well plates, as previously described^43^. The L6 cells were stained in triplicate wells with 100 nM Mitotracker Green FM for 30 min in serum-free media and fluorescence was measured with Cytation 3 Multi-Mode Reader (BioTek) with excitation wavelength 490 nm and emission 516 nm. The fluorescence was normalized with the protein content of the wells, which was measured using the BCA protein assay.

### Western Blotting

The cellular proteins were extracted using RIPA buffer and protein concentrations were measured by BCA protein assay kit. Triplicate protein samples were run in 4–12% NuPAGE Bis-Tris gels (Life Technologies, Espoo, Finland) and transferred to polyvinylidene fluoride (PVDF) membranes (GE Healthcare, Uppsala, Sweden). Membranes were blocked in 3–5% milk or BSA in TBS-Tween-20, washed with TBS-Tween-20 and incubated overnight at +4 °C with primary antibodies followed by washing with TBS-Tween-20 and incubation with secondary antibodies for 1 h at room temperature. The bands were visualized using enhanced chemiluminescence and images were captured with Image Quant RT-ECL system (GE Healthcare). Bands were quantified by Quantity One software (Bio-Rad, Hercules, CA, USA). The antibodies were from following sources: SIRT1 (07–131) was from Millipore. Phospho-AMPK (2535), AMPK (2532) and FOXO1 (2880) were from Cell Signaling Technology, Beverly, MA, USA. PGC1α (sc-13067), Ac-FKHR (acetylated FOXO1, sc-49437), NRF1 (sc-13031), GLUT4 (sc-1608) and donkey anti-goat IgG-HRP (sc-2020) were purchased from Santa Cruz. Anti-rabbit IgG-HRP (NA934V) and anti-mouse IgG-HRP (NA931V) were from GE Healthcare. α-tubulin (T5168) was from Sigma. The details of the antibody dilutions are shown in the Supplementary Table 1.

### ADP/ATP and NAD^+^/NADH assays

L6 myotubes were differentiated in in 6-well plates and treated with 60 µM compounds for 24 h in duplicate wells. ADP/ATP Ratio Assay kit (Abcam) and NAD^+^/NADH Ratio Assay kit (Abcam) were used according to the manufacturer’s instructions.

### Statistical analyses

Statistical differences between groups were tested using one-way analysis of variance (ANOVA) and Bonferroni post-hoc test, with *p* < 0.05 considered as statistically significant. Data analysis was performed using GraphPad Prism version 5.03 for Windows (GraphPad Software, La Jolla, CA, USA).

### Microarray Analysis

L6 myotubes were treated with 60 µM test compounds or DMSO for 24 h in differentiation media in three independent experiments, resulting in three replicate microarrays in each treatment group. Total RNAs were extracted with RNeasy Mini kit (Qiagen. Hilden, Germany) and RNA quality was assessed with the Agilent Bioanalyzer 2100 (Agilent Technologies, Espoo, Finland). Total RNA (200 ng) was converted to cDNA with AffinityScript RNase Block (Agilent Technologies), labelled according to manufacturer’s instructions (Agilent Technologies) and purified using RNeasy mini spin columns (Qiagen). RNA Spike-In Kit (Exiqon, Vedbaek, Denmark) was used to monitor the success of the labelling. Samples were then mixed with blocking agent, fragmentation and hybridization buffer, and were hybridized to Agilent SurePrint G3 Rat GE 8 × 60 K Microarrays for 17 h at 65 °C before washing and scanning with Agilent Scanner G2505C using manufacturer’s protocols. Agilent Feature Extraction software was used to extract data from raw microarray image files.

The data was processed with limma package of the Bioconductor software (http://www.bioconductor.org)^44^. Microarray data are MIAME compliant. Differentially expressed transcripts were analysed by Bayes moderated t-statistics followed by Benjamini-Hochberg’s correction method to control false discovery rate (FDR)^44,45^, with significance threshold set at *p* < 0.05. Heat map and clustering analysis of the transcripts were made with the R heatmap.2 function for Gene Ontology (GO) gene sets.

### Data availability

Microarray raw data have been deposited in the NCBI Gene Expression Omnibus (GEO), accession no. GSE90833). Other datasets generated during the current study are available from the corresponding author on reasonable request.

## Electronic supplementary material

Supplementary Information
