# Supplementary material for: Plant-derived compounds strigolactone GR24 and pinosylvin activate SIRT1 and enhance glucose uptake in rat skeletal muscle cells
Source: Sci Rep. 2017 Dec 14;7:17606. doi: 10.1038/s41598-017-17840-x (PMC5730588; doi:10.1038/s41598-017-17840-x)

Supplementary information

# Plant-derived compounds strigolactone GR24 and pinosylvin activate SIRT1 and enhance glucose uptake in rat skeletal muscle cells

**Shalem Modi[1], Nagendra Yaluri[1], Tarja Kokkola[1,+], Markku Laakso[1,2,*,+]**

[1]Institute of Clinical Medicine, Internal Medicine, University of Eastern Finland, 70210 Kuopio, Finland
[2]Department of Medicine, Kuopio University Hospital, 70210 Kuopio, Finland

*Corresponding author, markku.laakso@uef.fi
+Equal contribution

**Supplementary Figure 1**

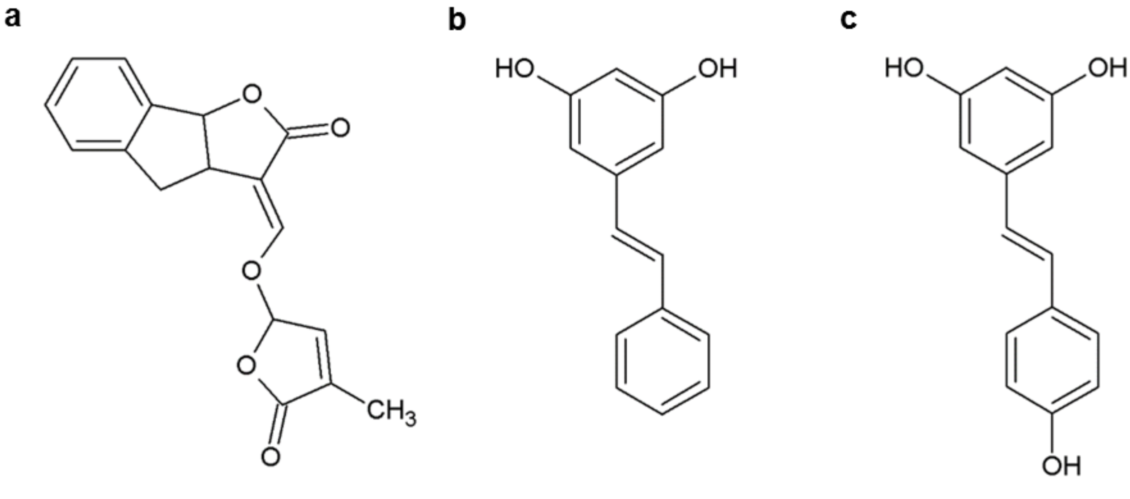


**Supplementary Figure 1.** Chemical structures of GR24 (a), pinosylvin (b) and resveratrol (c).

## Supplementary Figure 2

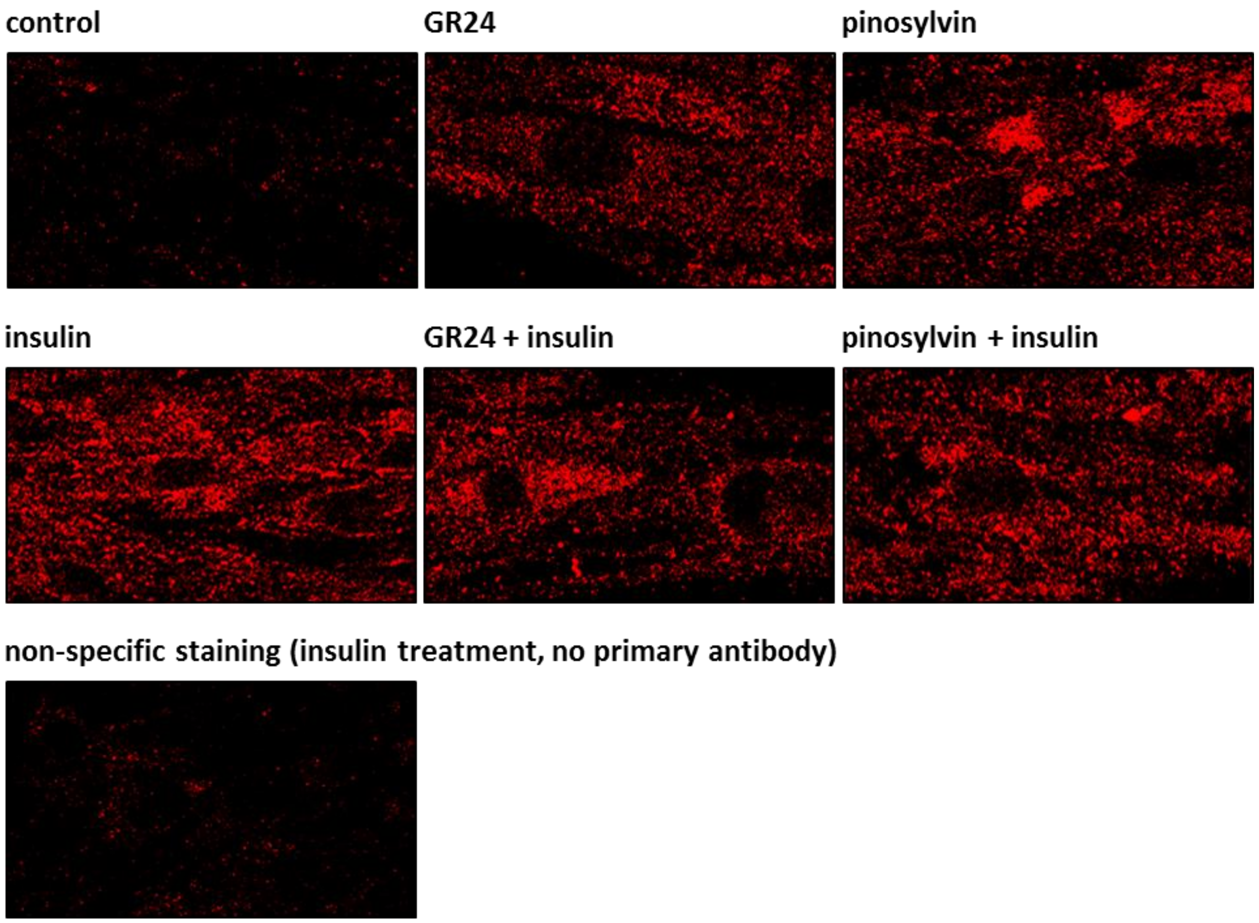


**Supplementary Figure 2.** Visualisation of cell surface GLUT4 by fluorescent microscopy. L6 myotubes were treated for 6 h with 60 µM test compounds or with DMSO, insulin was present when indicated. For the image showing the non-specific staining, the cell treatment was identical to the image above, but the primary antibody was omitted during the staining procedure.

**Supplementary Figure 3**

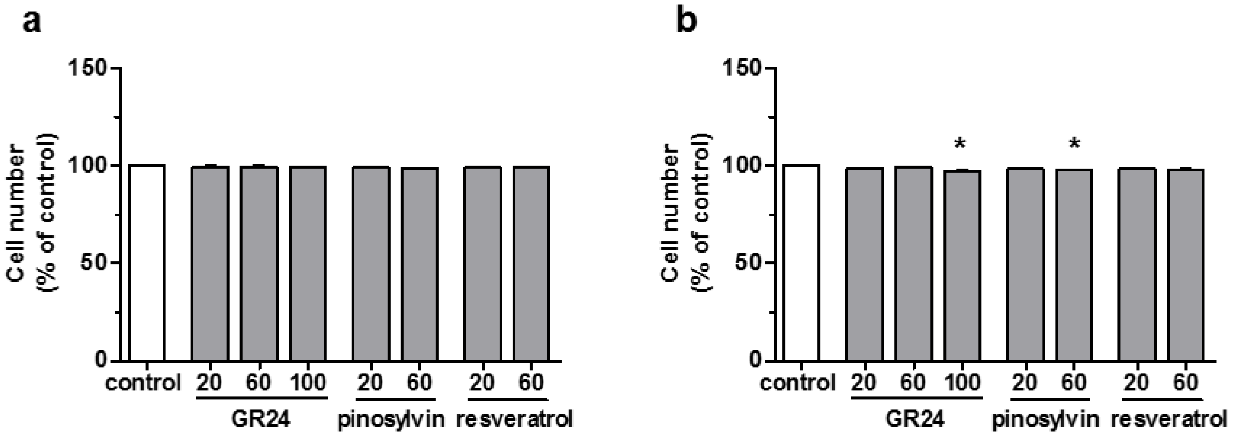


**Supplementary Figure 3.** Sulforhodamine B cytotoxicity/proliferation assays. L6 myotubes were treated with 20-100 µM test compounds (grey bars) or with DMSO (white bars) for 6 h (a) or 24 h (b). Bars represent the mean ± SEM of two independent experiments. *$p$ < 0.05 vs control.

**Supplementary Figure 4**

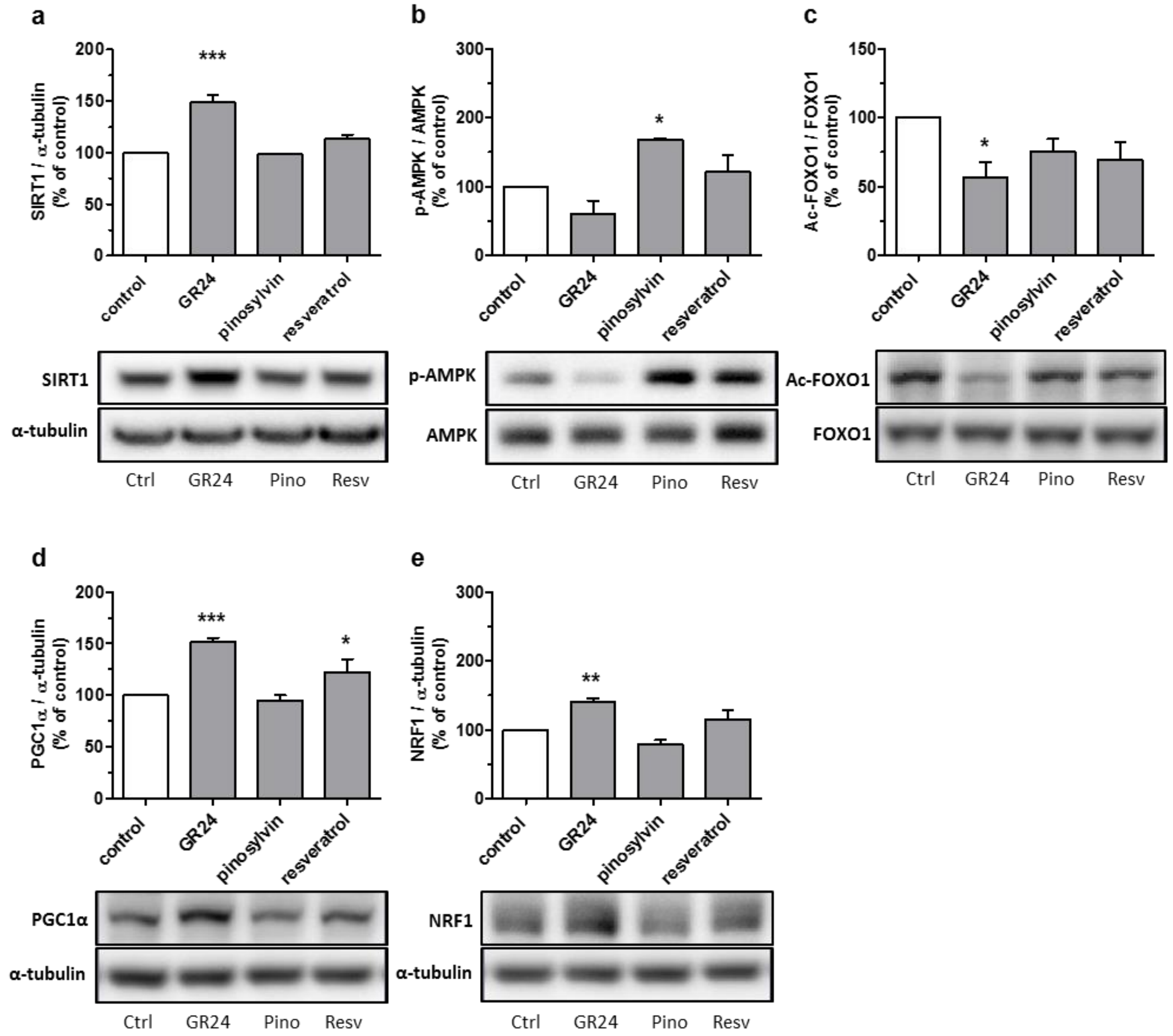


**Supplementary Figure 4.** The effects of GR24, pinosylvin and resveratrol in high glucose conditions. (a-e) L6 myotubes were treated with 60 µM test compounds (grey bars) or with DMSO (white bar) for 24 h in media containing 16.7 mM glucose. (a) SIRT1 expression was analysed by western blotting; bars represent the quantification results of SIRT1 normalised with α-tubulin. (b) AMPK phosphorylation was analysed by western blotting; bars represent the quantification results of phospho-AMPK normalised with total AMPK. (c) The acetylation level of the endogenous SIRT1 substrate FOXO1 was analysed by western blotting; bars represent the quantification results of acetylated FOXO1 normalised with total FOXO1. (d) PGC1α expression was analysed by western blotting; bars represent the quantification results of PGC1α normalised with α-tubulin. (e) NRF1 expression was analysed by western blotting; bars represent the quantification results of NRF1 normalised with α-tubulin. (a-d) Bars represent the mean ± SEM of three independent experiments. *$p < 0.05$, **$p < 0.01$ and ***$p < 0.001$ vs control. Representative western blots are shown below. Proteins in lower panels were detected from the same membranes after stripping and reprobing.

**Supplementary Figure 5**

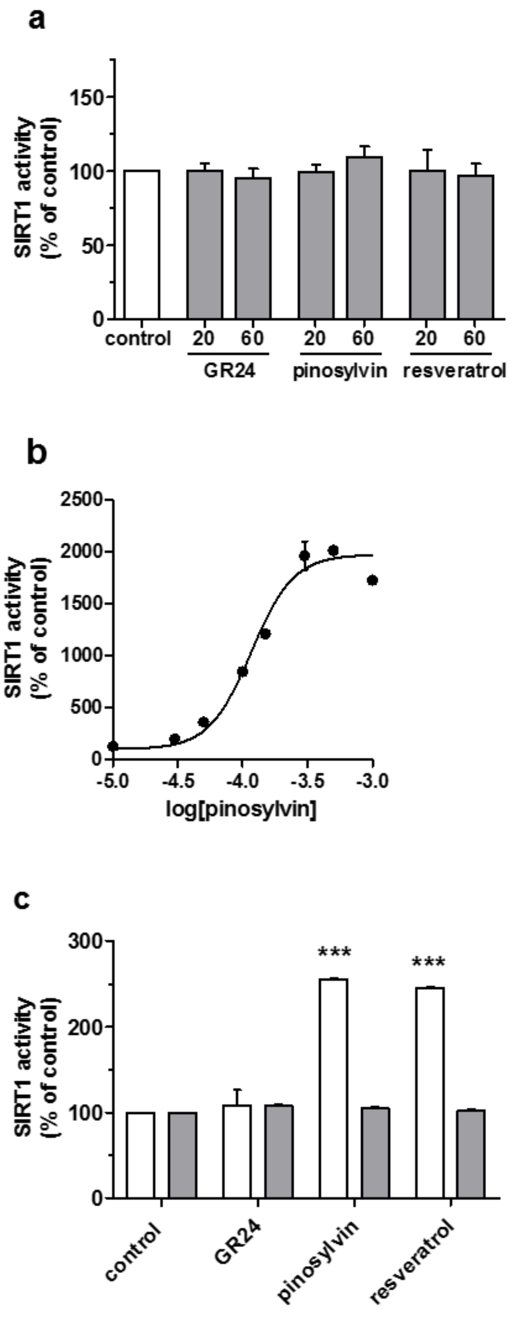


**Supplementary Figure 5.** GR24 and pinosylvin activate SIRT1 through different mechanisms. (a) SIRT1 activity in cell lysates analysed with Fluor de Lys *in vitro* activity assay. L6 myotubes were treated with 20 µM or 60 µM test compounds (grey bars) or with DMSO (white bar) for 24 h. Bars represent the mean ± SEM of three to four independent experiments. (b) Dose-response curve for pinosylvin in Fluor de Lys *in vitro* activity assay. Each point represents mean ± SEM of two independent experiments. (c) Recombinant SIRT1 activity in SIRTainty *in vitro* activity assay analysed using two different substrates: a fluorescently labelled Fluor de Lys assay substrate based on the sequence of p53 (white bars), and a proprietary non-labeled SIRTainty assay substrate (grey bars). The compounds were used at 100 µM concentration. Bars represent the mean ± SEM of two to four independent experiments. ***$p < 0.001$ vs control.

**Supplementary Figure 6**

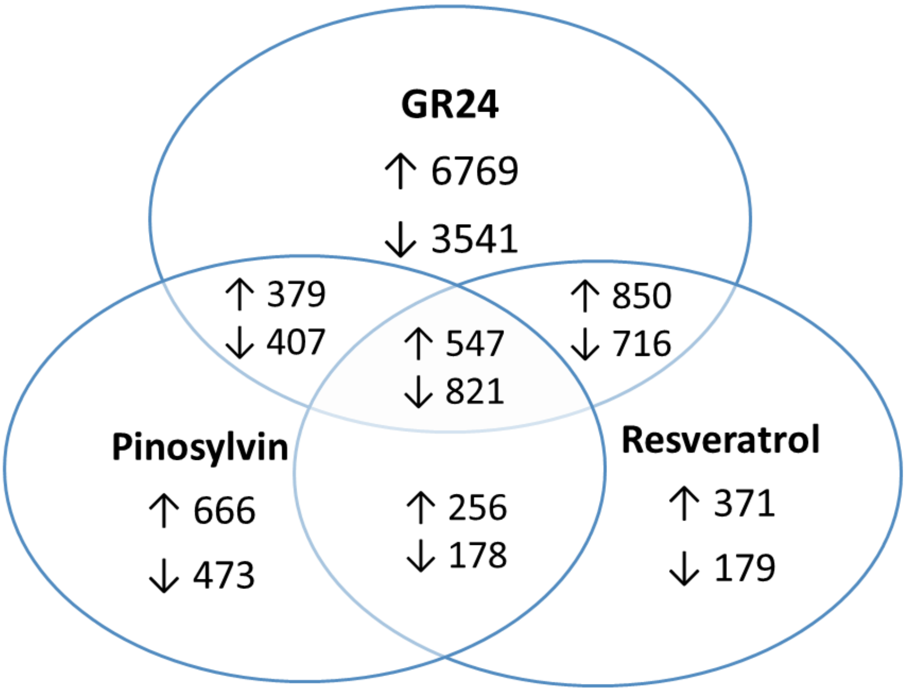


**Supplementary Figure 6.** Venn diagram showing the number of differentially expressed transcripts (↑ upregulated, ↓ downregulated) after exposure of L6 myotubes to 60 µM test compounds or control treatment for 24 h.

**Supplementary Figure 7**

## Mitochondrion_organization_and_biogenesis (GO)

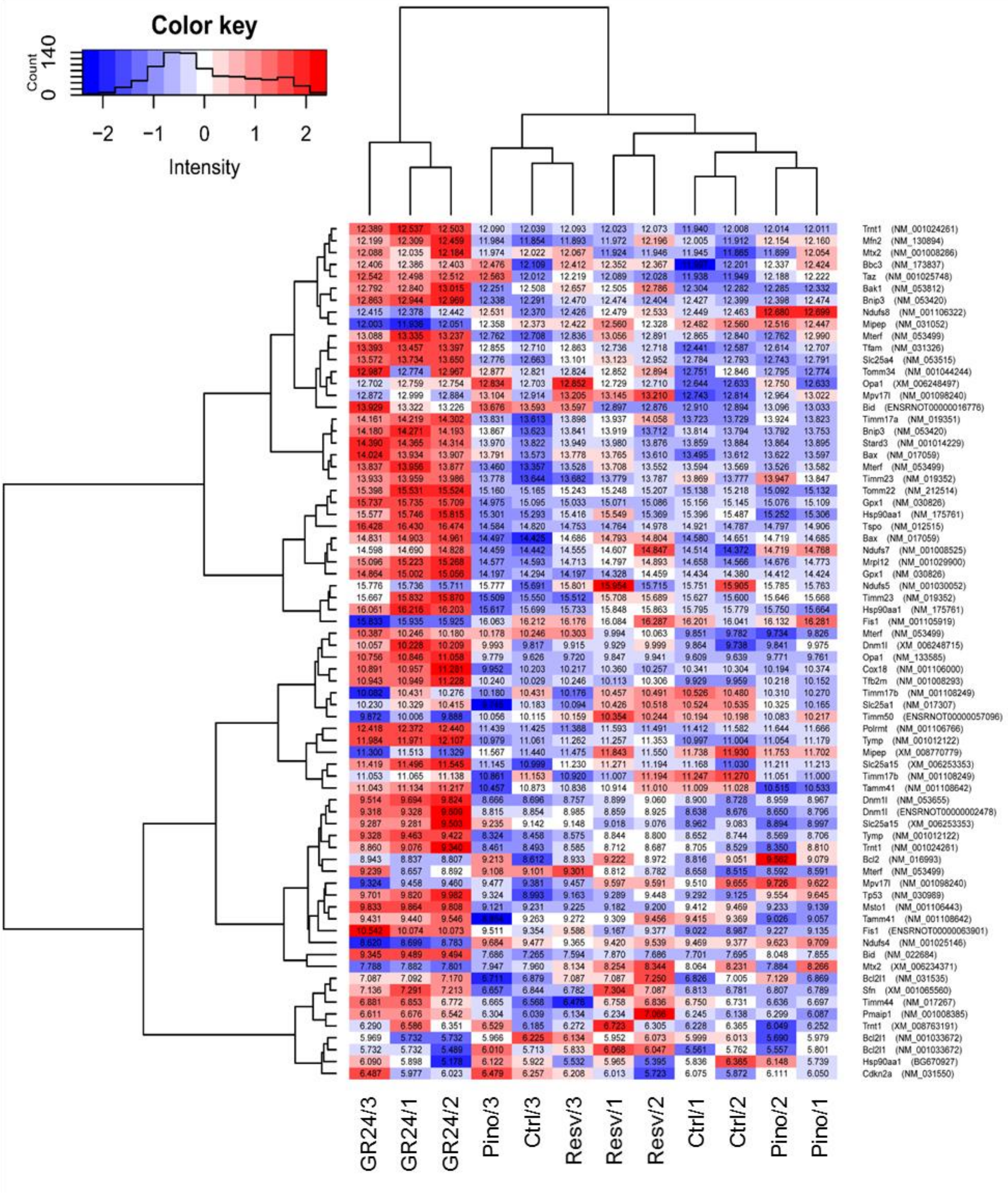


**Supplementary Figure 7.** Heat map and clustering analysis of the transcripts of the Gene Ontology gene set MITOCHONDRION_ORGANIZATION_AND_BIOGENESIS after exposure of L6 myotubes to 60 µM test compounds or control treatment for 24 h. Different lines represent different probes and different columns indicate different samples (3 per treatment group). Red colours indicate high fluorescence signals, while blue colours indicate low signals.

**Supplementary Figure 8**

## Insulin_receptor_signaling_pathway (GO)

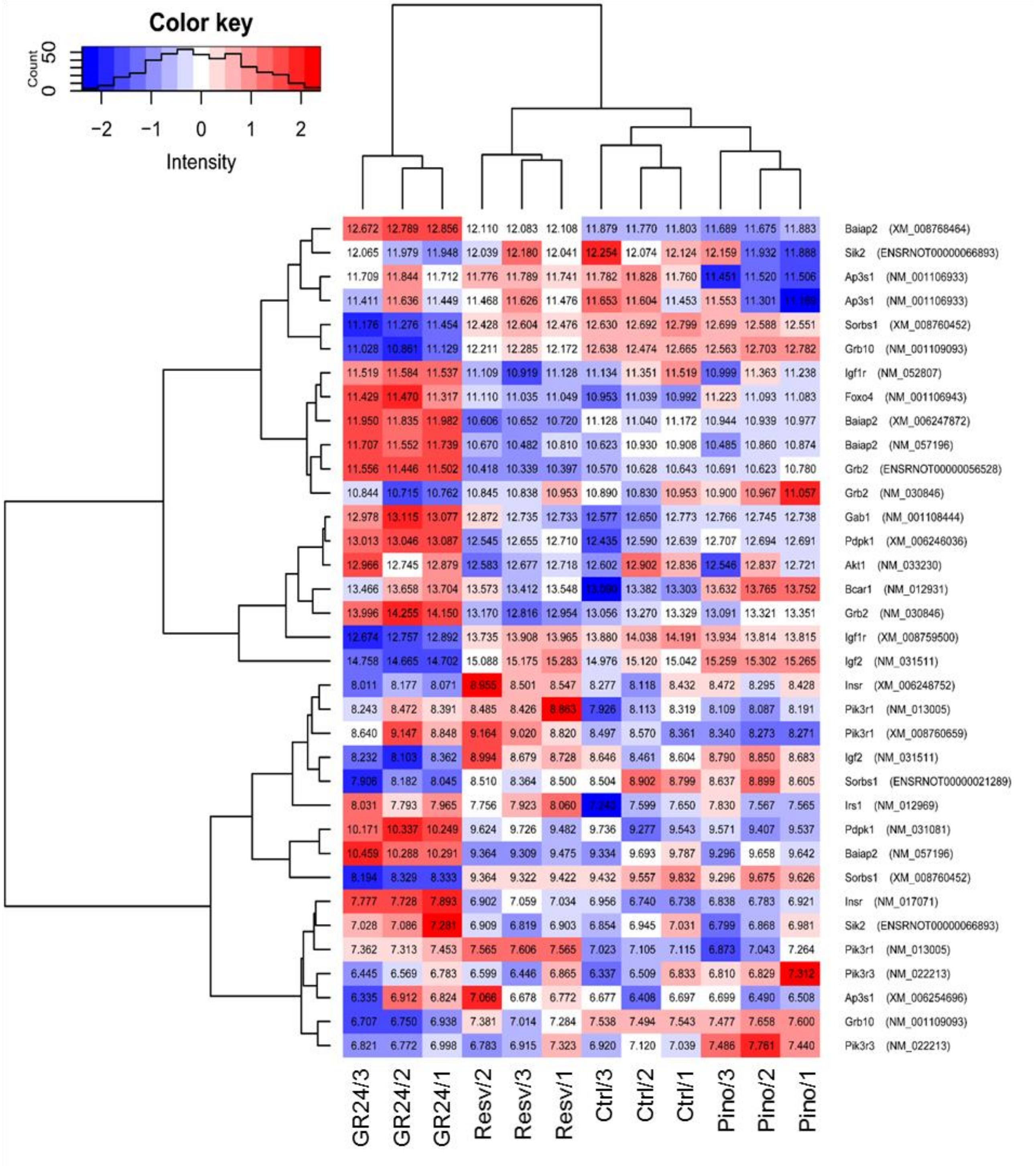


**Supplementary Figure 8.** Heat map and clustering analysis of the transcripts of the Gene Ontology gene set INSULIN_RECEPTOR_SIGNALING_PATHWAY after exposure of L6 myotubes to 60 µM test compounds or control treatment for 24 h. Different lines represent different probes and different columns indicate different samples (3 per treatment group). Red colours indicate high fluorescence signals, while blue colours indicate low signals.

**Supplementary Table 1.** Antibody dilutions

| Antibody | Product code | Dilution | Diluent |
|---|---|---|---|
| **Primary antibodies** | | | |
| SIRT1 | Millipore 07-131 | 1:3000 | 3% milk + 0.05% TBS-Tween-20 |
| AMPK | CST 2532 | 1:1000 | 5% BSA + 0.1% TBS-Tween-20 |
| Phospho-AMPK | CST 2535 | 1:1000 | 5% BSA + 0.1% TBS-Tween-20 |
| FOXO1 | CST 2880 | 1:1000 | 5% BSA + 0.1% TBS-Tween-20 |
| Ac-FKHR | Santa Cruz sc-49437 | 1:1000 | 5% BSA + 0.1% TBS-Tween-20 |
| PGC1α | Santa Cruz sc-13067 | 1:1000 | 5% BSA + 0.05% TBS-Tween-20 |
| NRF1 | Santa Cruz sc-13031 | 1:1000 | 5% milk + 0.05% TBS-Tween-20 |
| GLUT4 | Santa Cruz sc-1608 | 1:1000 | 5% BSA + 0.05% TBS-Tween-20 |
| α-tubulin | Sigma T5168 | 1:10000 | 3% milk in 0.05% PBS-Tween-20 |
| **Secondary antibodies** | | | |
| Anti-rabbit IgG-HRP (used with SIRT1 primary ab) | GE Healthcare NA934V | 1:40000 | 3% milk + 0.05% TBS-Tween-20 |
| Anti-rabbit IgG-HRP (used with AMPK & phospho-AMPK primary abs) | GE Healthcare NA934V | 1:15000 | 5% milk + 0.1% TBS-Tween-20 |
| Anti-rabbit IgG-HRP (used with FOXO1 & Ac-FKHR primary abs) | GE Healthcare NA934V | 1:10000 | 5% BSA + 0.1% TBS-Tween-20 |
| Anti-rabbit IgG-HRP (used with PGC1α primary ab) | GE Healthcare NA934V | 1:10000 | 5% BSA + 0.05% TBS-Tween-20 |
| Anti-rabbit IgG-HRP (used with NRF1 primary ab) | GE Healthcare NA934V | 1:10000 | 5% milk + 0.05% TBS-Tween-20 |
| Anti-goat IgG-HRP (used with GLUT4 primary ab) | Santa Cruz sc-2020 | 1:10000 | 5% BSA + 0.05% TBS-Tween-20 |
| Anti-mouse IgG-HRP (used with α-tubulin primary ab) | GE Healthcare NA931V | 1:20000 | 3% milk in 0.05% PBS-Tween-20 |

Full blot images for Fig. 1. Bands shown in Fig. 1 are indicated by red boxes.

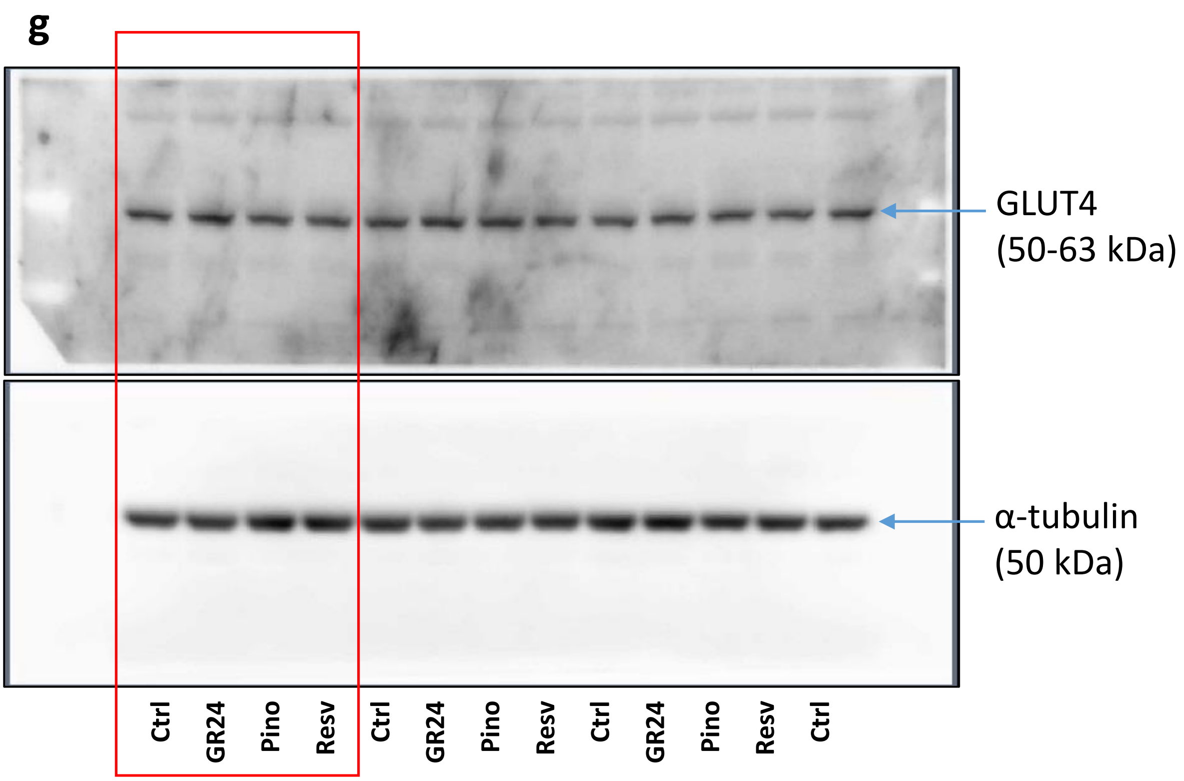


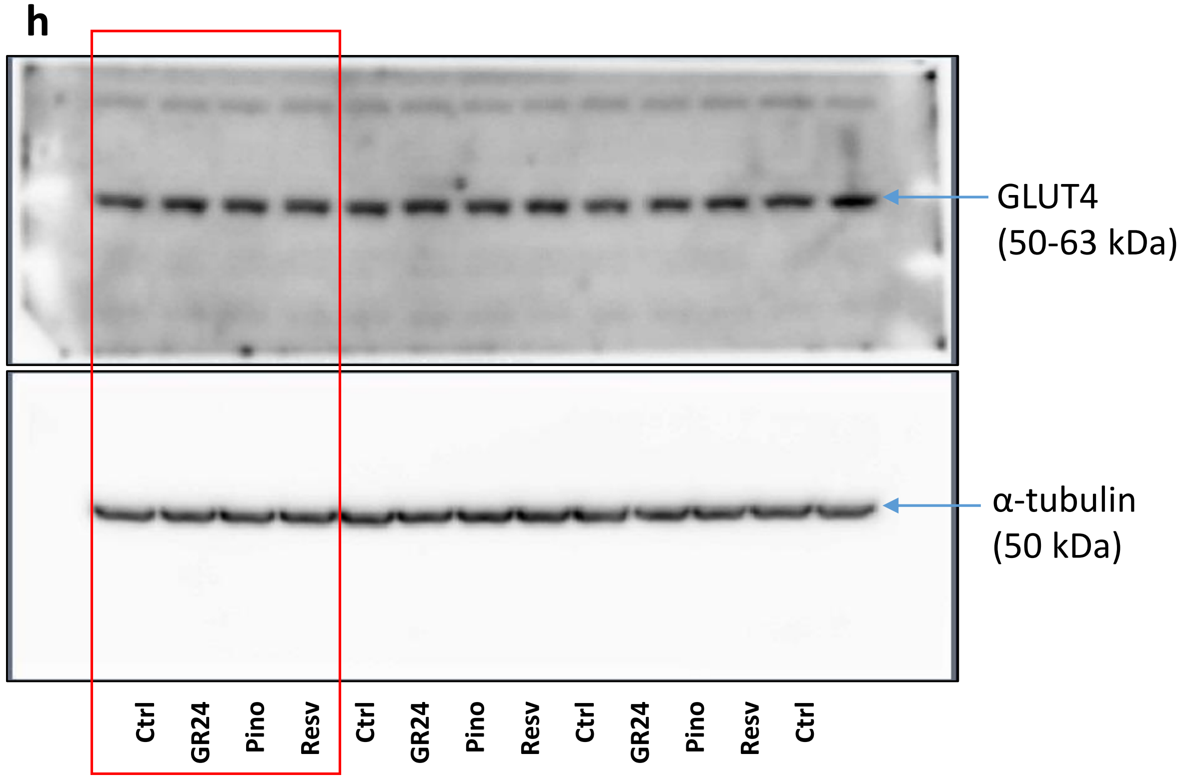

Full blot images for Fig. 2. Bands shown in Fig. 2 are indicated by red boxes.

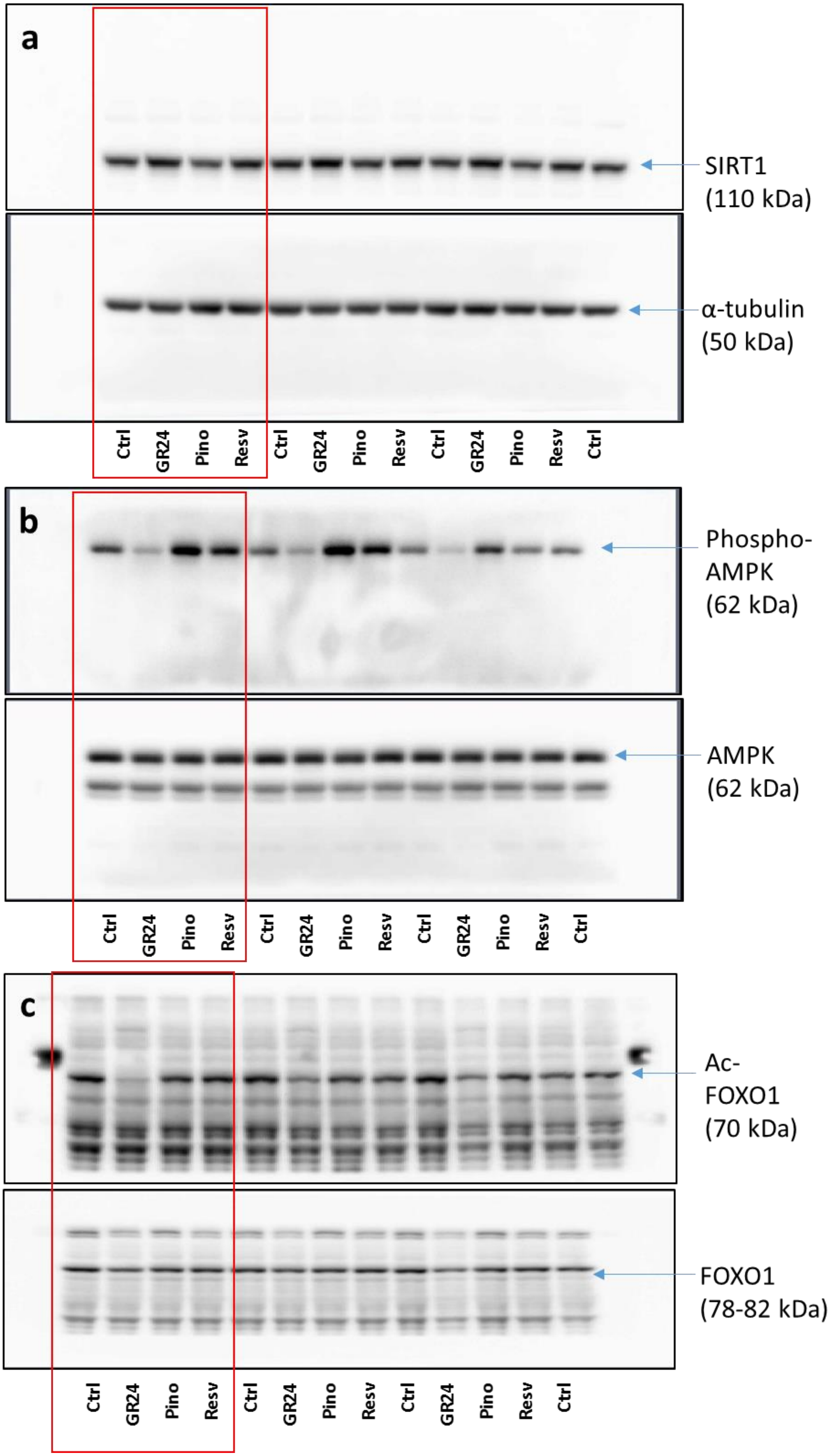

Full blot images for Fig. 3. Bands shown in Fig. 3 are indicated by red boxes.

e

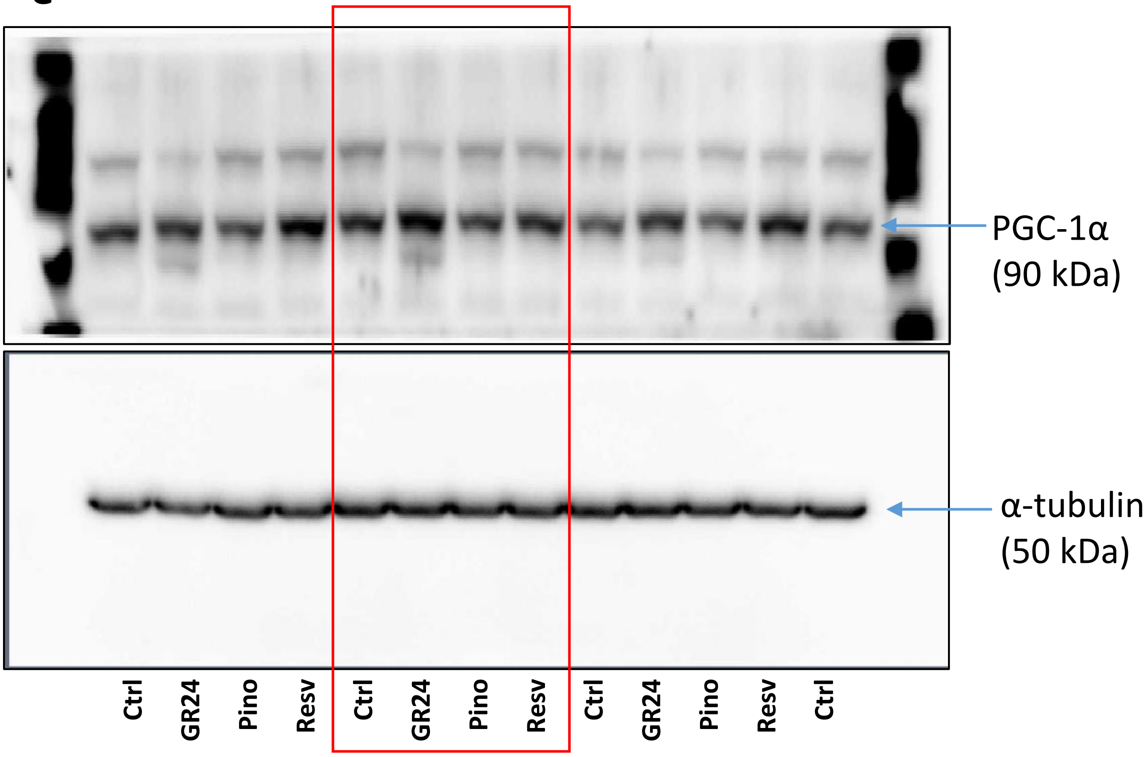


f

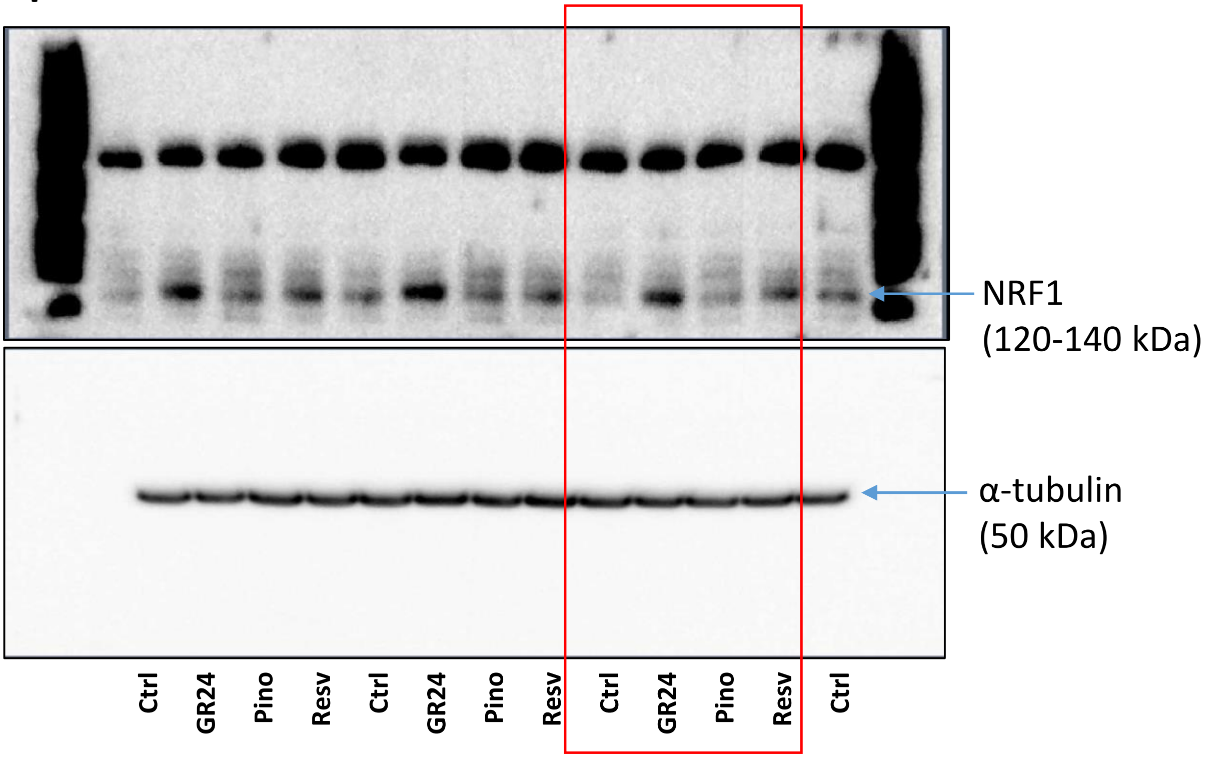

Full blot images for Supplementary Fig. 4a-c. Bands shown in Supplementary Fig. 4 are indicated by red boxes.

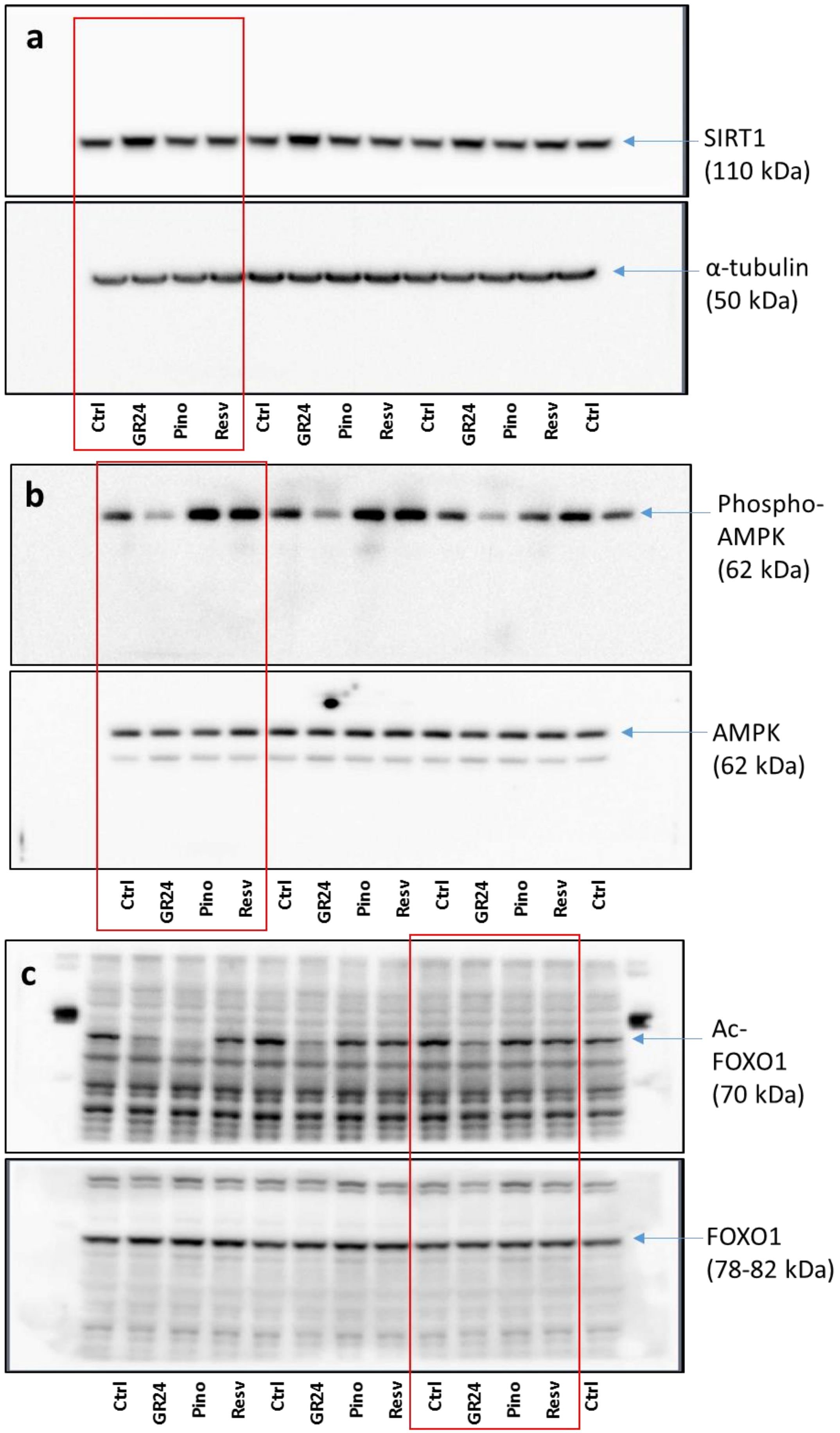

Full blot images for Supplementary Fig. 4d-e. Bands shown in Supplementary Fig. 4 are indicated by red boxes.

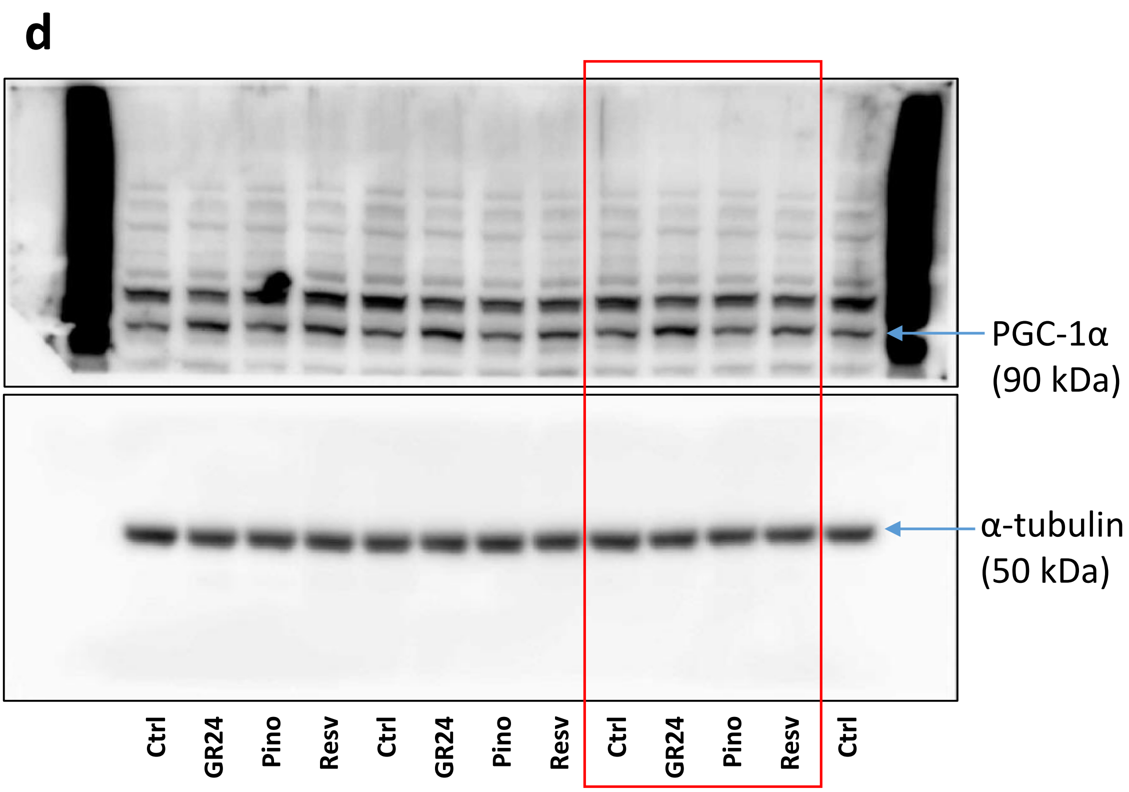


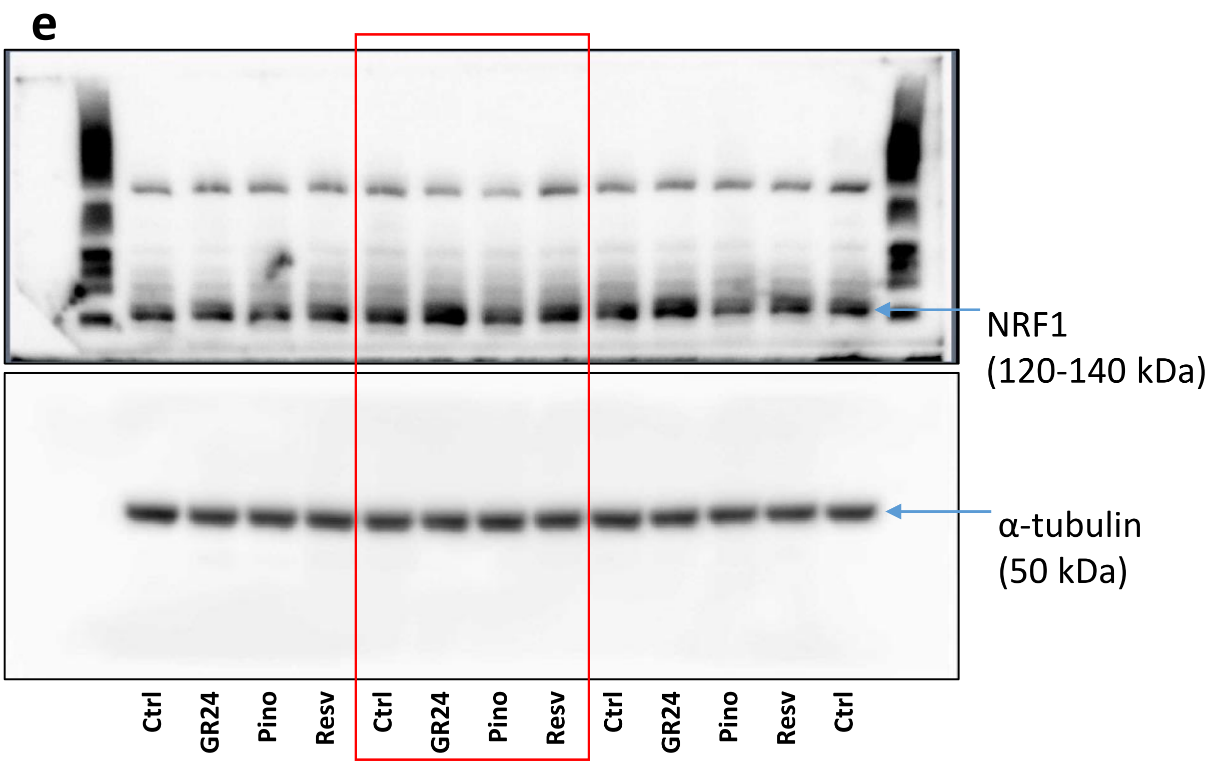

Supplement: Supplementary file 1 — Supplementary Information [file 41598_2017_17840_MOESM1_ESM.pdf]
